# Swine Inflammation and Necrosis Syndrome Is Associated with Plasma Metabolites and Liver Transcriptome in Affected Piglets

**DOI:** 10.3390/ani11030772

**Published:** 2021-03-11

**Authors:** Robert Ringseis, Denise K. Gessner, Frederik Loewenstein, Josef Kuehling, Sabrina Becker, Hermann Willems, Mirjam Lechner, Klaus Eder, Gerald Reiner

**Affiliations:** 1 Institute of Animal Nutrition and Nutrition Physiology, Justus Liebig University Giessen, Heinrich-Buff-Ring 26-32, 35392 Giessen, Germany; Robert.Ringseis@ernaehrung.uni-giessen.de (R.R.); Denise.Gessner@ernaehrung.uni-giessen.de (D.K.G.); Klaus.Eder@ernaehrung.uni-giessen.de (K.E.); 2 Department of Veterinary Clinical Sciences, Clinic for Swine, Justus Liebig University Giessen, Frankfurter Strasse 112, 35392 Giessen, Germany; Frederik.Loewenstein@lsz.bwl.de (F.L.); josef.kuehling@vetmed.uni-giessen.de (J.K.); sabrina.becker-2@vetmed.uni-giessen.de (S.B.); hermann.willems@vetmed.uni-giessen.de (H.W.); 3 UEG Hohenlohe, Am Wasen 20, 91567 Herrieden, Germany; mirjam.lechner@web.de

**Keywords:** inflammation, necrosis, swine, tail biting, liver transcriptome, plasma metabolome, lipopolysaccharide, microbial-associated molecular patterns, piglets

## Abstract

**Simple Summary:**

Swine inflammation and necrosis syndrome (SINS) is a newly identified syndrome associated with inflammatory and necrotic signs in different body parts of suckling piglets, weaners, and fatteners. While the proven inflammatory basis of the disease and the development of signs in even newborns indicate a primarily endogenous etiology, the mechanisms underlying SINS development are largely unknown. In this study, the hypothesis was tested that SINS is indirectly triggered by the translocation of gut-derived microbial components into the liver, thereby causing derangements of liver metabolism, by comparing the hepatic transcriptome and plasma metabolome of SINS-affected and unaffected piglets. Apart from demonstrating that hepatic inflammation occurs in SINS-affected piglets, the study clearly shows for the first time that several metabolic derangements are induced in SINS-affected piglets that may contribute to the clinical and histopathological signs of SINS.

**Abstract:**

Swine Inflammation and Necrosis Syndrome can lead to severe clinical signs, especially in tails, ears, teats, and claws in pigs. Clinical and histopathological findings in newborn piglets with intact epidermis indicate a primarily endogenous etiology, and microbial-associated molecular patterns (MAMPs), such as lipopolysaccharide (LPS) are assumed to play a central role in the development of the syndrome. We hypothesized that swine inflammation and necrosis syndrome (SINS) is indirectly triggered by gut-derived MAMPs entering the circulatory system via the liver and thereby causing derangements on liver metabolism. To test this hypothesis, metabolomes, candidate genes of the liver and liver transcriptomes of 6 piglets with high-grade clinical signs of SINS (SINS high) were examined and compared with 6 piglets without significant signs of SINS (SINS low). Several hepatic pro-inflammatory genes and genes involved in stress response were induced in piglets of the SINS high group. The most striking finding from hepatic transcript profiling and bioinformatic enrichment was that the most enriched biological processes associated with the approximately 220 genes induced in the liver of the SINS high group were exclusively related to metabolic pathways, such as fatty acid metabolic process. Within the genes (≈390) repressed in the liver of the SINS high group, enriched pathways were ribosome biogenesis, RNA processing, RNA splicing, spliceosome, and RNA transport. The transcriptomic findings were supported by the results of the metabolome analyses. These results provide the first evidence for the induction of an inflammatory process in the liver of piglets suffering from SINS, accompanied by lipid metabolic derangement.

## 1. Introduction

Swine inflammation and necrosis syndrome (SINS) is a newly identified syndrome that can lead to significant clinical alterations in suckling piglets [1], weaners, and fatteners [2]. Signs of SINS can be inflammation and necrosis at the tail, ears, heels, claw coronary bands, teats, umbilicus, vulva, and face and are associated with inflammatory signs at the pathohistological level. Especially on exposed body parts, such as claws, heels, and soles, the degree of SINS and environmental effects such as unfavorable floor conditions influence each other [2]. But even in animals with macroscopically intact epidermis, considerable prevalence of vasculitis, intima proliferation, and thrombosis of the vessels were detected [2,3], including newborn piglets immediately after birth in which signs could not result from biting or technopathy [3]. Based on EFSA consensus statement, inflammation and necroses can seriously impair animal welfare [4,5]. Injuries to the skin, tail, and ears are generally used as valuable characteristics for assessing animal welfare in pigs. Therefore, SINS is likely to be a relevant diagnostic indicator of impaired animal welfare in swine.

The proven inflammatory basis of the disease, the development of signs in newborns who have not been exposed to bites or technopathies, and the involvement of different body parts in the syndrome, such as tail, ear, claws, and teats, indicate a primarily endogenous etiology. Circulatory disorders in the tail of suckling piglets as an etiology of necroses have already been suspected in earlier studies [6,7,8], and have recently been proven based on the histopathological evidence of vasculitis and thrombosis at the tail base proximal to the lesions in affected piglets [3]. The circulatory disorder was also clinically shown by a rapid decrease in temperature within this part of the tail base of affected piglets measured by thermal imaging camera [9]. We therefore suggest that the local inflammatory processes in the vicinity of the blood vessels are causative for SINS development. Various authors showed associations between plasma concentrations of deoxynivalenol (DON) and lipopolysaccharides (LPS) from the sow and necroses on the tail, ears, or coronary bands of the piglets [10,11,12]. In line with this, Nordgreen et al. [13] recently proposed that problems in the areas of microbiota, gut barrier, housing environment and hygiene, immune activation, mycotoxins, psychological stress, nutritional status and feed composition can synergistically lead to inflammation directly or indirectly in association with LPS. An important source for the flooding of LPS and other microbial components, collectively termed microbial-associated molecular patterns (MAMPs), is the intestine (for overview see [12]), from which MAMPs are translocated into the liver via the portal vein. Since the liver acts like a firewall, which is able to degrade MAMPs owing to the ability of liver-resident macrophages (Kupffer cells) and other intrahepatic immune cells [14,15], the liver largely prevents the entry of MAMPs into the systemic circulation. However, in the case that the gut barrier is disrupted (“leaky”), the liver is confronted with high levels of MAMPs. In this case, MAMPs are recognized not only by hepatic immune cells but also by hepatic parenchymal cells which are abundantly equipped with specific MAMP recognition receptors, such as Toll-like receptors (TLR). Upon sensing of MAMPs by TLRs, various inflammatory and stress-signaling pathways, such as nuclear factor-kappa B (NF-κB), c-JUN N-terminal kinase (JNK), and endoplasmic reticulum (ER) stress-induced unfolded protein response (UPR) are stimulated, thereby, causing hepatic inflammation and an impaired functionality of the organ [16].

In view of this, we hypothesized that SINS is indirectly triggered by gut-derived MAMPs entering the liver and the circulatory system, thereby causing derangements in liver metabolism [17,18,19,20]. In order to characterize these metabolic derangements of SINS piglets for the first time, the hepatic transcriptome and plasma metabolome of piglets with high-grade SINS was compared with that of piglets with either no or only low-grade signs of the syndrome.

## 2. Materials and Methods

### 2.1. Ethical Statement

The study was approved by the authorities in Stuttgart, Baden-Wurttemburg, Germany with file numbers 35–9185.81/0415 and 35–9185.64/0035.

### 2.2. Animals and Experimental Design

The animal experiment was carried out in the conventional stables of the State Institution for Swine Breeding (Landesanstalt für Schweinezucht, LSZ, Baden-Württemburg, Boxberg, Germany). Total of 12 out of 120 piglets from 40 sows of a herd of Baden-Württemburg Genetics hybrid sows artificially inseminated with Pietrain semen were used for the experiment. The complete experiment was described by Reiner et al. [2]. From all piglets scored for SINS in this experiment, six piglets with least (SINS low) and six piglets with highest (SINS high) SINS-Scores were selected for transcriptomics.

### 2.3. Clinical Scoring

Piglets were clinically scored for SINS on the third day of life as described by Reiner et al. [2]. Owing to time constraints and to reduce the animal load, clinical signs were recorded with a digital camera (Lumix, Panasonic Corporation, Osaka, Japan). Later, images were detailly evaluated using Windows Media Player (Version 12, Microsoft GmbH, Munich, Germany) according to a standardized protocol. The tail base and tip, ears, teats and navel, coronary bands, wall horn, balls and soles of the feet along with the face were all initially assessed individually with regard to the below mentioned clinical characteristics. Clinical characteristics were scored with either 0 (=not visible sign) or 1 (=visible sign). A semiquantitative score was used in the case that different degrees or values had to be validated. The tail was scored for swelling (0/1), redness (0/1), rhagades (0/1), exudation (0/1), bleeding (0/1), tail necrosis (0/1), and annular constriction of tail tissue (0/1). The tail base was separately screened and scored for the presence of bristles (0 = present; 1 = absent), swelling of the tail base (0/1), redness of the tail base (0/1), exudation (0/1) and clinical signs of necrosis (0/1). Ears were scored for the presence of bristles (0 = present; 1 = absent), congested ear veins (0/1), and necrosis of the ears (0/1). Teats were scored for scab formation (0/1), swelling (0/1), reddening (0/1), necrosis (0/1), and congested blood vessels (0/1). The navel was scored for clinical signs of inflammation (0/1). The face was scored for edema around the eyes (0/1) and nasal oedema (0/1). Each claw was individually scored for wall layering (0/1), wall bulging (0/1), wall bleeding (0/1), sole reddening (0/1), detachment of sole from heel (0/1), reddening of heel (0/1), heel bleeding (0/1), detachment of heel (0/1), redness of coronary band (0/1), exudation of coronary band (0/1), and necrosis of coronary band (0/1). In the end, mean scores of the eight claws, resp. 4 feet were used for the calculation of the SINS-Score. Congestion of the inner thigh veins was also recorded (0/1). All individual findings were summarized to the SINS-Score without weighting. Scores between 0 and 34 were theoretically possible.

### 2.4. Sample Collection

After the clinical scoring, the piglets were euthanized and sampled. The piglets were anaesthetized intramuscularly with a mixture of 40 mg/kg ketamine (Ursotamin^®^ 10%, Serumwerk Bernburg AG, Bernburg, Germany) and 4 mg/mL azaperone (Stresnil^®^, Elanco, Indianapolis, IN, USA). This was followed by collection of 4.5 mL serum (Monovette Serum 4.5 mL, Sarstedt AG, Nümbrecht, Germany) from the cranial vena cava using a 1.2 × 4.0 mm cannula (Agani, Terumo Corporation, Tokyo, Japan) for the metabolom study. For the use of the S-Monovettes^®^, a multi-adapter (Sarstedt AG) with Luer-Lock system was used to attach the cannulae. The animals were euthanized immediately after blood collection with an intracardiac injection of 45 mg/kg pentobarbital sodium (Release^®^ 500 mg/mL, WDT Eg, Garbsen, Germany). Death was determined by auscultation of the heart with a stethoscope (double-headed stethoscope, Henry Schein GmbH, Melville, NY, USA), the absence of spontaneous respiration, and by testing the eyelid closure, cornea, nasal septum and interclaw reflexes.

The animals were placed on a stainless steel table (work table type KST-041, R&S Edelstahl und Technik GmbH, Hamm, Germany) with an absorbent pad (sick pad, Henry Schein GmbH). After death, the abdominal cavity of the animals was opened along the linea alba with a scalpel blade (Aesculap^®^ BB522, B.Braun AG, Melsungen, Germany). Approximately 1 g of material was removed from the liver at the lobus sinister medialis.

Aliquots of the liver were excised, washed in sterile ice-cold NaCl solution (0.9%) and collected in microcentrifuge tubes (Sarstedt, Nümbrecht, Germany). The liver samples were immediately snap-frozen in liquid nitrogen and stored at −80 °C, pending RNA extraction.

### 2.5. RNA Extraction, Microarray Hybridization, and Bioinformatic Analysis

Total RNA from liver aliquots (20 mg) of six suckling piglets each of the SINS low group (either no or only low-grade signs of SINS) and the SINS high group (high-grade signs of SINS) was extracted using TRIzol reagent (Invitrogen, Karlsruhe, Germany) according to the manufacturer’s protocol. RNA quantity and quality were determined spectrophotometrically and by electrophoresis as described in detail [21]. The average RNA concentration and RNA integrity number (RIN) of all total RNA samples (n = 12, means ± SD) was 454 ± 34 ng/µL and 8.0 ± 0.6, respectively. For microarray hybridization, RNA samples were sent on dry-ice to the Center of Excellence for Fluorescent Bioanalytics (Regensburg, Germany) and, following a further RNA quality check using an Agilent 2100 Bioanalyzer (RIN of all samples: 7.7 ± 0.5), processed using an Affymetrix GeneChip Porcine Gene 1.0 Sense Target array, which covers 19,212 genes represented by 394,580 probes, according to the manufacturer’s instructions as described [21]. Following scanning of the processed GeneChips, cell intensity files, which provided a single intensity value for each probe cell, were generated from the image data using the Command Console software (Affymetrix). Correction of background and normalization of probe cell intensity data was carried out using the Robust Multichip Analysis (RMA) algorithm with the Expression Console software (Affymetrix). RMA algorithm is a log scale multi-chip analysis approach fitting a robust linear model at the probe level to minimize the effect of probe-specific affinity differences. Expression levels of transcripts are measured using log transformed perfect match values, after carrying out a global background adjustment and across microarray normalization [22]. Annotation of microarrays was performed with the NetAffx annotation file “Porcine Annotations, CSV format, Release 36 (6.4 MB, 4/13/16)”. The microarray data of this study have been provided in the NCBI’s Gene Expression Omnibus public repository ([23]; “GSE167489”). Owing to the rather moderate differences in the hepatic transcriptomes between group SINS high and group SINS low, transcripts were defined as differentially expressed when the fold change (FC) between group SINS high vs. group SINS low was >1.3 or <−1.3 and the *p*-value of the unpaired Student’s *t*-test (two-tailed distribution, two-sample equal variance) was <0.05. Identical or similar filter criteria were also applied in several recent studies [21,24,25], in which treatment-induced changes of the transcriptome were only moderate and the application of more stringent filter criteria (e.g., false discovery rate and/or FC > 2.0 or <−2.0) failed to filter a substantial number of genes being sufficient to perform gene set enrichment analysis (GSEA). In the present study, filtering of differentially expressed transcripts using the Benjamini & Hochberg false discovery rate adjustment method could not be applied, because the adjusted *p*-values for all transcripts were >0.05.

GSEA was carried out with the list of differentially expressed transcripts in order to identify enriched Gene Ontology (GO) biological process terms using the freely available DAVID 6.8 bioinformatic resource [26,27]. Biological process terms were considered as enriched if *p* < 0.05. GSEA was carried out separately for the up- and down-regulated transcripts, respectively, for easier interpretation of data. According to this approach, biological processes identified as enriched within up-regulated and down-regulated genes are assumed to be activated and inhibited, respectively.

### 2.6. Validation of Microarray Data Using qPCR Analysis

Microarray data of 30 strongly regulated mRNAs (15 up-regulated, 15 down-regulated) were validated by qPCR. Synthesis of cDNA and qPCR analysis was performed with a Rotor-Gene Q system (Qiagen, Hilden, Germany) using gene-specific primer pairs (Eurofins MWG Operon, Ebersberg, Germany) as described recently in detail [28]. Primer pairs were designed using Primer3 [29] and Basic Local Alignment Search Tool [30]. Characteristics of primer pairs are shown in Supplementary file 1: Table S1. Normalization was carried out using multiple reference genes (ACTB, RPS9, SDHA) as described recently [31]. The mean value calculated from normalized individual values of group SINS low was set to 1 and mean and SD of group SINS high were scaled proportionally.

### 2.7. Targeted Metabolite Screening

Plasma concentrations of specific metabolites from six compound classes [acylcarnitines (n = 40), amino acids (n = 21), hexoses (n = 1), glycerophospholipids (n = 90), sphingolipids (n = 15), amino acid metabolites (n = 21)] were determined using Absolute*IDQ* p180 kit by Biocrates (Innsbruck, Austria).

### 2.8. Statistics

Prevalence of clinical signs and differences between piglets with low and high SINS scores were assessed with the IBM-SPSS program package (version 26) using chi2 test. If there were less than 5 cases in a group, Fisher’s exact test was used. All other data were evaluated by Student’s *t* test using the Minitab statistical software (Release 13, Minitab Inc., State College, PA, USA).

## 3. Results

### 3.1. Clinical SINS Scores and Organ Alterations

According to the selection criteria for the piglets to be assigned to the SINS low or SINS high group, both differed significantly in the SINS score achieved (Table 1). The extreme animals of the SINS high group had a SINS score of 12, while the animals of the SINS low group had a minimum SINS score of 0.83. In the piglets of the SINS high group, with the exception of the face, navel and claw wall, all animals were marked by clinical findings on all parts of the body examined. In most cases, the organ changes were characterized by bristle loss, swelling, and redness. Exudation and necrosis occurred in fewer animals. Such alterations were particularly present at the tail, teats, and coronary bands with low prevalence. In contrast, the piglets in the SINS low group did not show any clinical changes on the tail, ears, face, claws, and soles. In some piglets, only reddened teats, combusted veins at the hind limb, alterations of the coronary bands and heels were noticeable. Thus, alterations in piglets of the control group only occurred in particularly exposed parts of the body.

### 3.2. Expression of Genes Involved in Inflammation and Stress Response in the Liver of Piglets of the SINS Low Group and the SINS High Group

**Figure 1:**
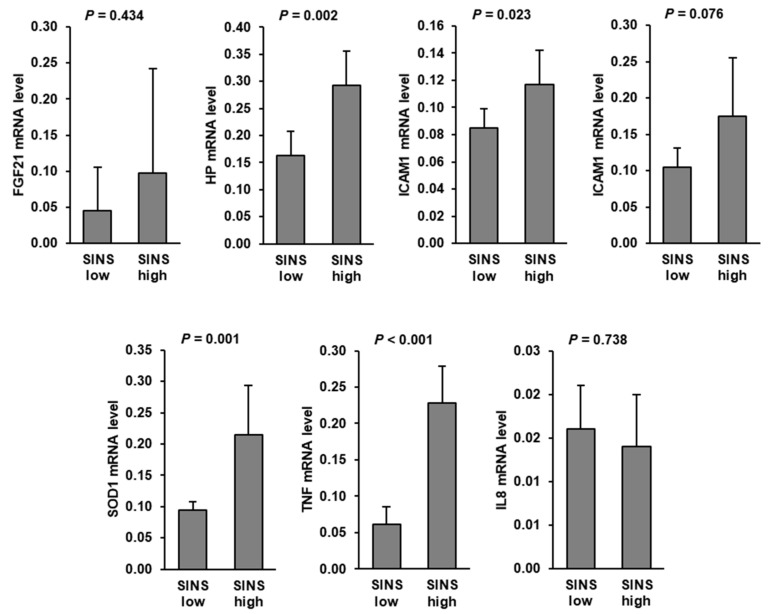
Relative mRNA levels of genes involved in inflammation and stress response in the liver of suckling piglets of the SINS high group and the SINS low group. Data are means ± SD for n = 6 piglets per group.

Hepatic mRNA levels of TNF, SOD1, HP, and ICAM1 were significantly increased in suckling piglets of the SINS high group compared to those of the SINS low group. In addition, hepatic mRNA level of IL6 tended (*p* < 0.1) to be increased in the SINS high group compared to the SINS low group. No differences were found between groups regarding hepatic mRNA levels of FGF21 and IL8 (Figure 1).

### 3.3. Identification of Differentially Expressed Transcripts in the Liver of Piglets between the SINS High Group and the SINS Low Group

**Figure 2:**
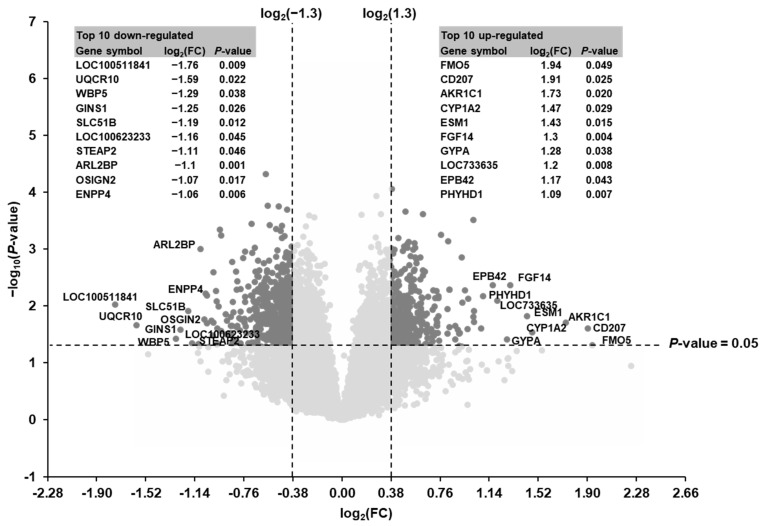
Volcano plot illustrating the differentially regulated transcripts in the liver of suckling piglets between the SINS high group and the SINS low group. The double filtering criteria are indicated by horizontal (*p*-value < 0.05) and vertical (FC > 1.3 or FC < −1.3) dashed lines. Transcripts in the upper left and the upper right corner represent the down-regulated and the up-regulated transcripts, respectively. The top 10 up- and down-regulated transcripts are indicated with their gene symbols and log2(FC) and *p*-values are presented in the tables in the left (down-regulated) and right (up-regulated).

Considering the two filter criteria (FC > 1.3 or <−1.3; *p* < 0.05) a total of 615 transcripts out of 19,212 transcripts screened were identified as differentially expressed (up-regulated: 223, down-regulated: 392) in the liver of suckling piglets between the SINS high group and the SINS low group (Figure 2). The top 10 up-regulated transcripts were in decreasing order of their FCs: FMO5, CD207, AKR1C1, CYP1A2, ESM1, FGF14, GYPA, LOC733635, EBP42, and PHYHD1. The top 10 down-regulated transcripts were in increasing order of their FCs: LOC100511841, UQCR10, WBP5, GINS1, SLC51B, LOC100623233, STEAP2, ARL2BP, OSGIN2, and ENPP4. The log_2_(FC) and the *p*-value of the top 10 up- and down-regulated genes are also shown in Figure 2. The log_2_(FC), FC, and *p*-value of all differentially expressed transcripts are listed in Supplementary file 1: Table S2.

### 3.4. Technical Validation of Microarray Data

Microarray data of 30 differentially expressed transcripts were validated by qPCR. As shown in Table 2, the effect direction (positive or negative log_2_(FC)) was the same between microarray and qPCR for all validated transcripts. As expected, the effect size (value of the log_2_(FC)) differed to some extent for the validated transcripts between microarray and qPCR. Statistical analysis of qPCR data revealed that 14 and 8 of the validated transcripts were regulated either significantly (*p* < 0.05) and tended to be regulated (*p* < 0.1), respectively, whereas 8 of the transcripts were not regulated (*p* > 0.1).

### 3.5. Identification of Biological Processes and Pathways Affected by the Differentially Expressed Transcripts in the Liver of Piglets between the SINS High Group and the SINS Low Group

**Figure 3:**
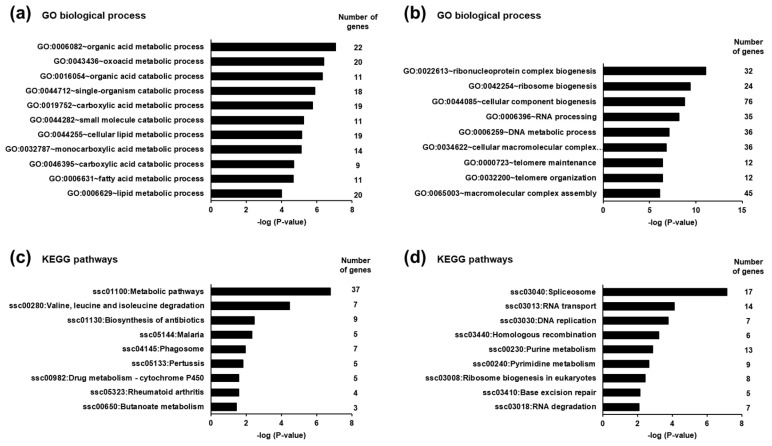
Most enriched gene ontology (GO) biological process terms (**a**,**b**) and KEGG pathways (**c**,**d**) associated with the genes up-regulated (**a**,**c**) and down-regulated (**b**,**d**) in suckling piglets between the SINS high group and the SINS low group. GO terms and KEGG pathways are sorted by their enrichment *p*-values (EASE score) (top: lowest *p*-value, bottom: highest *p*-value). (**a**) Only GO terms with *p*-values < 0.0001 are shown. (**b**) Only GO terms with *p*-values < 0.000001 are shown. (**c**) Only KEGG pathways with *p*-values < 0.05 are shown. (**d**) Only KEGG pathways with *p*-values < 0.01 are shown.

To identify biological processes and pathways affected by the differentially expressed transcripts in the liver of piglets between the SINS high group and the SINS low group, GSEA was performed using GO biological process terms and KEGG pathways, respectively. GSEA of the transcripts up-regulated in suckling piglets of the SINS high group revealed that the most enriched biological process terms were exclusively related to the metabolism of organic acids, such as oxoacids and carboxylic acids including fatty acids. Examples of the most enriched (lowest *p*-value) terms are organic acid metabolic process, oxoacid metabolic process, and organic acid catabolic process. The biological process terms with *p*-values < 0.01 including the number of genes assigned to these terms are shown in Figure 3a. GSEA of the transcripts down-regulated in the suckling piglets of the SINS high group demonstrated that the most enriched biological process terms were mainly dealing with ribosome biogenesis, RNA processing, RNA splicing, and telomere organization. Owing to the large number of identified GO terms only those with *p*-values < 0.0001 including the number of genes assigned to these terms are presented in Figure 3b.

The most enriched KEGG pathways assigned to the transcripts up-regulated in the suckling piglets of the SINS high group were metabolic pathways and valine, leucine, and isoleucine degradation. All KEGG pathways with a *p*-value < 0.05 including the number of genes assigned to these terms are shown in Figure 3c. The most enriched KEGG pathways assigned to the transcripts down-regulated in the suckling piglets of the SINS high group were spliceosome, RNA transport, and DNA replication. All KEGG pathways with a *p*-value < 0.05 including the number of genes assigned to these terms are shown in Figure 3d.

### 3.6. Concentrations of Plasma Metabolites in Suckling Piglets of the SINS High Group and the SINS Low Group

The plasma concentrations of 21 amino acids and 15 amino acid metabolites, except ornithine, carnosine, methionine sulfoxide, and taurine, did not differ between the two groups (Supplementary file 1: Tables S3 and S4). The plasma concentration of methionine sulfoxide (Met-SO) was higher (*p* < 0.05) and that of ornithine, carnosine, and taurine tended to be higher (*p* < 0.1) in the SINS high group than in the SINS low group.

Plasma concentrations of carnitine and acyl-carnitines, lysophosphatidylcholine species, and sphingomyelin species were not different between the two groups (Supplementary file 1: Tables S5–S7). In addition, plasma concentration of hexoses did not differ between groups (SINS low: 3670.3 ± 645.6 µmol/L, SINS high: 4103.5 ± 963.5 µmol/L; n = 6/group).

Several differences between the two groups were found with regard to the plasma concentrations of phosphatidylcholine (PC) species (Supplementary file 1: Table S8). Among the diacyl (aa) PC species, the plasma concentrations of C36:6 and C40:3 were elevated (*p* < 0.05) and those of C36:4 and C38:6 tended to be elevated (*p* < 0.1) in the SINS high group compared to the SINS low group. Among the acyl-alkyl (ae) PC species, the plasma concentrations of C34:1, C38:0, and C42:3 were higher (*p* < 0.05) and those of C30:0, C30:1, C32:1, C36:0, C36:3, C36:4, C38:5, and C40:1 tended to be higher (*p* < 0.1) in the SINS high group than in the SINS low group. The sum of total aa PC species, the sum of total ae PC species, and the sum of total aa and ae PC species tended to be elevated in the SINS high group compared to the SINS low group (*p* < 0.1).

### 3.7. Correlations between Significantly Altered Plasma Metabolites and the Most Strongly Regulated Hepatic Genes in Suckling Piglets

**Figure 4:**
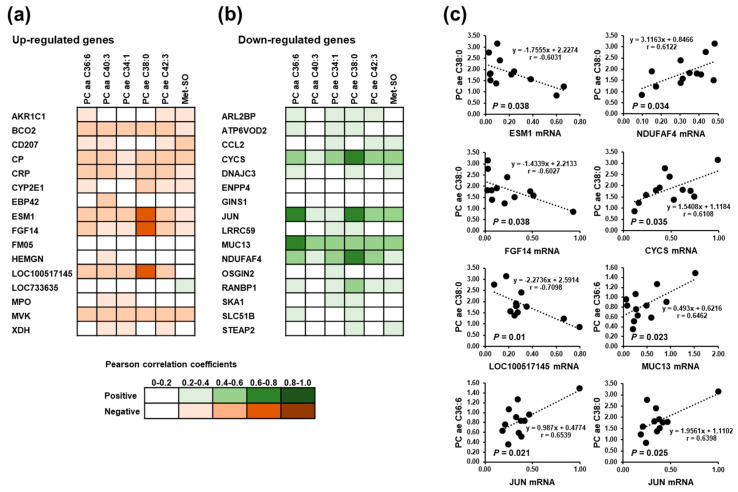
Pearson correlation coefficients from correlation analysis between significantly altered plasma metabolites and the most strongly up-regulated (**a**) and down-regulated (**b**) hepatic genes in the suckling piglets. (**c**) Linear regression for the significant correlations.

In order to investigate the statistical relationships between altered hepatic genes expression and significantly altered plasma metabolites (PC aa C36:6, PC aa C40:3, PC ae C34:1, PC ae C38:0, PC ae C42:3, Met-SO), regression analysis was carried out. Among the up-regulated genes, the correlations as indicated by Pearson correlation coefficients (r) between hepatic gene expression level and plasma metabolite concentration was weak (r < 0.6), except PC ae C38:0 (Figure 4). Plasma concentration of PC ae C38:0 was found to be negatively correlated with the hepatic expression of ESM1 (r = −0.6031; *p* = 0.038), FGF14 (r = −0.6027; *p* = 0.038), and LOC100517145 (r = −0.7098; *p* = 0.01) (Figure 4). Similarly, among the down-regulated genes correlations between hepatic gene expression and plasma metabolite concentrations were weak (r < 0.6), except PC ae 36:6, which was positively correlated with hepatic gene expression of JUN (r = 0.6539; *p* = 0.021) and MUC3 (0.6462; *p* = 0.023), and PC ae C38:0, which was positively correlated with hepatic gene expression of CYCS (r = 0.6108; *p* = 0.035), JUN (r = 0.6398; *p* = 0.025), and NDUFAF4 (r = 0.6122; *p* = 0.034) (Figure 4).

## 4. Discussion

In line with the recently raised assumption of a central role of an inflammatory condition triggered by MAMPs, such as LPS, in the development of SINS [1,2,3,9], the present study provides the first evidence for the induction of an inflammatory process in the liver of piglets with high-grade SINS. This was evident from the observation that several hepatic pro-inflammatory genes and genes involved in stress response, such as TNF, HP, ICAM1, and SOD1 according to qPCR data and CRP according to microarray data, were induced in piglets of the SINS high group compared to those of the SINS low group. Since the piglets of this study were scored for clinical signs of SINS as early as the third day of life, our observations suggest that the inflammatory condition in the liver develops already intrauterine. Histo-pathological results of granulocytes, macrophages, and lymphocytes in the area of the inflammatory alterations at the tail of newborn SINS piglets directly after birth [3], support this assumption. Macrophages appear only after 2 to 3 days and lymphocytes only after at least 4 days [32] following the triggering insult. It can therefore be concluded that the trigger for the inflammation at the base of the tail must have occurred well before birth. Recently, it was demonstrated in mice that LPS injected into the intrauterine space gains access to the maternal-fetal compartment and interacts with the cells expressing functional TLR4, a receptor sensing MAMPs, in the placenta and fetus [33]. Regarding positive associations between plasma LPS concentration of sows and severity of SINS symptoms of the piglets [10,11,13], it is therefore likely that the hepatic inflammatory process in SINS-affected piglets was caused by MAMPs.

Following our hypothesis that metabolic derangements in the liver of SINS-affected piglets occur as a consequence of MAMPs entering the liver, we compared the hepatic transcriptome and plasma metabolome of piglets with high-grade SINS with that of piglets with either no or only low-grade signs of the syndrome. The most striking finding from hepatic transcript profiling and bioinformatic enrichment analysis using gene ontology category “biological process” was that the most enriched biological processes associated with the approximately 220 genes induced in the liver of the SINS high group were exclusively related to metabolic pathways, such as fatty acid metabolic process, cellular lipid metabolic process, carboxylic acid metabolic process, oxoacid metabolic process, and organic acid metabolic process. This was confirmed by gene set enrichment analysis using KEGG pathways demonstrating that the most enriched KEGG pathway associated with the genes induced in the SINS high group was the term metabolic pathways. The strong involvement of genes dealing with fatty acid and lipid metabolism in the liver of piglets of the SINS high group was reflected by the induction of malic enzyme 3 (ME3), insulin induced gene 1 (INSIG1), lipin 3 (LPIN3), acetyl-CoA acyltransferase 1 (ACAA1), acyl-CoA dehydrogenase, C-2 to C-3 short chain (ACADS), acyl-CoA synthetase medium-chain family member 3 (ACSM3), aldehyde dehydrogenase 5 family member A1 (ALDH5A1), glutaryl-CoA dehydrogenase (GCDH), hydroxyacid oxidase 2 (HAO2), and isovaleryl-CoA dehydrogenase (IVD). In contrast to the hepatic genes induced in the SINS high group, enrichment analysis using gene ontology category “biological process” and KEGG pathways within the hepatic genes repressed (≈390) in the SINS high group revealed ribosome biogenesis, RNA processing, RNA splicing, spliceosome, and RNA transport as the most enriched biological processes and pathways. While the specific metabolic consequence of these findings is less clear, it shows that SINS generally affects biological processes which play a role in gene transcription, thus explaining that SINS is associated with an altered hepatic transcriptome. Future studies are necessary to unravel the relevance of these findings.

In order to clarify if the SINS-induced alterations of metabolic pathways observed at the level of hepatic transcriptome was associated with changes at the level of metabolome, the plasma metabolome of the piglets was screened for changes between the SINS high group and the SINS low group using targeted analysis of several important classes of metabolic compounds. While piglets of both groups did not differ with regard to the plasma concentrations of amino acids, biogenic amines, acylcarnitines, hexoses, and sphingolipid species at that age, changes between groups were found regarding a number of specific lipid species, which are in line with our results from hepatic transcriptomic analysis that the genes induced in the SINS high group were preferentially involved in metabolic processes dealing with fatty acid metabolism. Namely, the diacyl PC species, C36:6 and C40:3, and the acyl-alkyl PC species, C34:1, C38:0, and C42:3, were significantly elevated in the plasma of piglets of the SINS high group. In addition, several diacyl (C36:4 and C38:6) and acyl-alkyl (C30:0, C30:1, C32:1, C36:0, C36:3, C36:4, C38:5, and C40:1) PC species in the plasma tended to be higher in piglets of the SINS high group than in those of the SINS low group. Although the precise meaning of these changes in plasma lipid species with regard to SINS is unknown, it is well documented that chronic liver diseases with an inflammatory component, such as non-alcoholic steatohepatitis, are associated with profound changes of fatty acid composition and total phospholipid content in the liver [34,35,36]. Such changes are likely explained by the existence of multiple crosstalk between inflammatory signaling pathways, such as NF-κB and UPR, and hepatic lipid metabolic pathways [37]. Interestingly, correlation analysis between altered lipid metabolites and hepatic transcript levels revealed positive correlations between the acyl-alkyl PC species 36:6 and 38:0 and hepatic transcript level of JUN, a well-known pro-inflammatory transcription factor encoding Jun proto-oncogene, AP-1 transcription factor subunit. Even though it is no proof of a causal link between hepatic inflammation and hepatic lipid metabolic derangements in SINS, our observation is at least indicative of an association between both symptoms. Further proof is required to substantiate this association.

## 5. Conclusions

In conclusion, the present study provides the first evidence that hepatic inflammation and lipid metabolic derangements occur in the liver of piglets with high-grade SINS compared with piglets with either no or only low-grade signs of the syndrome.

## Figures and Tables

**Table 1:** Prevalence of clinical signs in different body parts in piglets of the swine inflammation and necrosis syndrome (SINS) low group and the SINS high group.

Body Part	Clinical Signs	SINS Low	SINS High	*p*-Value
Tail base	no bristles	0.0	100.0	0.001
	swelling	0.0	100.0	0.001
	redness	0.0	16.7	
	exudation	0.0	16.7	
	necrosis	0.0	0.0	
	any	0.0	100.0	0.001
Tail	swelling	0.0	67.7	0.03
	scab formation	0.0	83.3	0.008
	rhagades	0.0	67.7	0.03
	exudation	0.0	100.0	0.001
	necrosis	0.0	16.7	
	annular constriction of tail tissue	0.0	0.0	
	bleeding	0.0	0.0	
	loss	0.0	0.0	
	any	0.0	100.0	0.001
Ears	no bristles	0.0	33.3	
	veins combusted	0.0	83.3	0.008
	necrosis	0.0	0.0	
	any	0.0	100.0	0.001
Face	eye edema	0.0	33.3	
	nasal edema	0.0	16.7	
	any	0.0	33.3	
Teats	veins combusted	0	33.3	
	swelling	0	83.3	0.008
	redness	16.7	33.3	
	necrosis	0	16.7	
	any	16.7	100.0	0.008
Thighs	veins combusted	33.3	83.3	
Navel	inflammation	50.0	33.3	
Coronary bands	redness	50.0	100.0	
	exudation	66.7	100.0	
	necrosis	0.0	50.0	
	any	66.7	100.0	
Claw wall	bulging	0.0	88.3	0.008
	bleeding	0.0	0.0	
	any	0.0	88.3	0.008
Soles	redness	0.0	100.0	0.001
	detachment	0.0	16.7	
	any	0.0	100.0	0.001
Heels	swelling	100.0	66.7	
	redness	83.3	83.3	
	cracks	16.7	0.0	
	detachment	0.0	0.0	
	any	100.0	100.0	
SINS-Score		0.8 ± 0.6	12.0 ± 1.2	<0.001

**Table 2:** qPCR validation of selected microarray data.

Gene Symbol	log_2_(FC) SINS High vs. SINS Low		*p*-Value	
	Microarray	qPCR	Microarray	qPCR
*FMO5*	1.94	1.37	0.049	0.067
*AKR1C1*	1.73	1.57	0.020	0.017
*ESM1*	1.43	2.38	0.015	0.016
*FGF14*	1.30	2.47	0.004	0.017
*AKR1C2*	1.20	0.90	0.008	0.097
*EPB42*	1.17	1.52	0.004	0.037
*HEMGN*	1.02	1.70	0.012	0.110
*CRP*	0.97	1.28	0.025	0.053
*BCO2*	0.93	1.10	0.009	0.040
*C3*	0.91	0.93	0.030	0.080
*MPO*	0.90	1.79	0.026	0.086
*XDH*	0.88	1.48	0.023	0.009
*CYP2E1*	0.87	1.51	0.039	0.012
*MVK*	0.77	0.93	0.001	0.001
*CP*	0.75	1.23	0.040	0.011
*GINS1*	−1.25	−0.93	0.026	0.054
*SLC51B*	−1.19	−1.46	0.012	0.022
*PCLAF*	−1.16	−0.85	0.045	0.120
*STEAP2*	−1.11	−0.86	0.046	0.134
*ARL2BP*	−1.10	−0.88	0.001	0.029
*ENPP4*	−1.06	−0.48	0.006	0.278
*DNAJC9*	−1.04	−0.69	0.020	0.077
*SKA1*	−1.00	−0.30	0.033	0.487
*NDUFAF4*	−1.00	−0.49	0.003	0.153
*CCL2*	−0.93	−0.40	0.028	0.363
*MUC13*	−1.01	−1.37	0.018	0.085
*ATP6V0D2*	−1.00	−0.58	0.020	0.085
*RANBP1*	−0.98	−0.70	0.005	0.067
*CYCS*	−0.94	−0.55	<0.001	0.250
*LRRC59*	−0.86	−0.79	0.046	0.123

## Data Availability

The microarray data have been deposited in MIAME compliant format in the NCBI´s Gene Expression Omnibus public repository “GSE167489”. The other datasets used and analyzed during the current study are available from the corresponding author on reasonable request.

## Supplementary Materials

The following are available online at https://www.mdpi.com/2076-2615/11/3/772/s1. Table S1: Characteristics of gene-specific primers. Table S2: Fold change (FC) and p-value of all differentially expressed transcripts. Table S3: Plasma concentrations of amino acids in suckling piglets of the SINS high group and the SINS low group. Table S4: Plasma concentrations of biogenic amines and other amino acid metabolites in suckling piglets of the SINS high group and the SINS low group. Table S5: Plasma concentrations of carnitine and acylcarnitines in suckling piglets of the SINS high group and the SINS low group. Table S6: Plasma concentrations of lysophosphatidylcholine (LPC) species in suckling piglets of the SINS high group and the SINS low group. Table S7: Plasma concentrations of sphingomyelin (SM) and hydroxy sphingomyelin (SM (OH)) species in suckling piglets of the SINS high group and the SINS low group. Table S8: Plasma concentrations of diacyl (aa) and acyl-alkyl (ae) phosphatidylcholine (PC) species in suckling piglets of the SINS high group and the SINS low group.
