# Supplementary material for: Swine Inflammation and Necrosis Syndrome Is Associated with Plasma Metabolites and Liver Transcriptome in Affected Piglets
Source: Animals (Basel). 2021 Mar 11;11(3):772. doi: 10.3390/ani11030772 (PMC8001383; doi:10.3390/ani11030772)
Supplement: Supplementary file 1 [file animals-11-00772-s001.pdf]

Supplementary file 1

Table S1 Characteristics of gene-specific primers

Gene	Forward primer (from 5` to 3`)<br>Reverse primer (from 5` to 3`)	PCR product size (bp)	NCBI GenBank Accession no.	Annealing temperature
Reference genes				
ACTB	GACATCCGCAAGGACCTCTA<br>ACATCTGCTGGAAGGTGGAC	205	XM_003124280	60
ATP5G1	CAGTCACCTTGAGCCGGGCGA<br>TAGCGCCCCGGTGGTTTGC	94	NM_001025218	64
GAPDH	GTCGGAGTGAACGGATTTGG<br>CCACTCCGGACACGCTTGCA	218	NM_001206359	59
RPS9	GTCGCAAGACTTATGTGACC<br>AGCTTAAAGACCTGGGTCTG	325	XM_003356050	60
HAS2	GAAAAGGCTAACCTACCCTG<br>TGTTGGACAAGACCAGTTGG	218	NM_214053	60
Target genes				
AKR1C1	TACAAGCCCGTCTGCAACCA<br>GCGAAGGGCAATTACGGCTG	225	NM_001044569	60
AKR1C2	ACCTTGCTCCTCCTACACAATG<br>TCAGGTGCATACGTGCCAAA	207	NM_001044570	60
ARL2BP	GCTGTCCTTCTCTTCCGCCT<br>GCCGCCATGTTAAACCCAGG	146	XM_003126968	60
ATP6V0D2	GACCCTCTACCCAACCTGTGG<br>ATGTCTTTCCCCCACTGCCAT	180	XM_003125581	60
BCO2	AATGCCTTTGAGGACCAGGG<br>TCAGCCTGTTTCACAGCACT	248	XM_003129854	60
C3	GCCACATCCTACGCCCTCTT<br>GGTACATGGTCACCACCGACA	352	NM_214009	60

CCL2	CTGCACCCAGGTCCTTGC<br>GACCCACTTCTGCTTGGGTTC	199	NM_214214	60
CP	GGTGGGAGACACCATCCGAG<br>GTGAGGCAGAAGGAGGCACA	155	NM_001267694	60
CRP	GAAGCTGTCACTGTGTCTCCTGG<br>AGTAAAGGCCGTGAGTGGTTTGG	154	NM_213844	60
CYCS	ACAAGGTCAGGCTGTTAGAATGC<br>GCTGATGGTACAGCTCCCCTA	144	NM_001129970	60
CYP2E1	TCTCCATCTGGAAGCACATC<br>TAGTGGAGCAAGACCTCCTT	220	NM_214421	60
DNAJC9	TGCTGGAGAGGTCCCATCCTAT<br>CCACTCCTTCATCAAGCCCCA	137	XM_003483498	60
ENPP4	TCGACCCGGCAGACTACACT<br>CCGTGGTCACCTAGTTTGAGGG	247	XM_001924940	60
EPB42	GGCATTTCCTGTGGTCAAGTGG<br>TGCTCGGCATTGTTTCTCGC	103	XM_013993102	60
ESM1	CCTTCGGGATGGAATGCAAGC<br>CACCCAGGATCAGCGTGGA	245	XM_013984770	60
FGF14	AACAGGAATCTGGTAGAGCCTGG<br>GTTCTCGGTACATGGCAACTTCC	135	XM_001924895	60
FGF21	CGATACCTCTACACGGATGA<br>CGTTGTAGCCATCCTCAAGA	262	NM_001163410	60
FMO5	TGATCCAGCCCTTAGGTGCC<br>GTATGGCGTTGGCTGTCCAC	169	XM_001928594	60
GINS1	AGGCTTGGACATCACACAGGAT<br>GCAATGGGCGAGTGAGGTTATG	213	XM_003483911	60
HP	GTTCGCTATCACTGCCAAAC<br>CAGTTTCTCTCCAGTGACCT	108	NM_214000	60
HEMGN	GCCTGAGGACTGTGCATCTG<br>CTCTGGTTCTTCTGCTTTGGGT	250	XM_021066411	60
ICAM1	CGGTGGCAGCCGTGGCTATC<br>TTGATGCAGCCCCGCTCGTC	208	NM_213816	60

IL6	AAGGTGATGCCACCTCAGAC<br>TCTGCCAGTACCTCCTTGCT	151	NM_001252429	60
IL8	ACTTCCAAACTGGCTGTTGC<br>GGAATGCGTATTTATGCACTGG	120	NM_213867	60
LRRC59	AGCCTCAGCGACCTGAATGA<br>CCACAGAACTCCGACGGTAGA	113	NM_001244951	60
MPO	ACTTTACCCCTGAGCCTGCC<br>CGTTGGTTCTTGATGCGGGG	130	XM_003131655	58
MUC13	TGTGTGTTGCTTTGGGTCCAG<br>CACAGCCAACTCCACTGTAGC	171	NM_001105293	60
MVK	CTGTGGCCTTGAACTTGAGA<br>CTGCTGGATGCCAATGTTTG	92	XM_001929184	60
NDUFAF4	AGTTGAAACCTTCCCCTGCTCC<br>CCTTTGGCTTCTGATGTGCTCC	204	XM_001925477	60
PCLAF	GGAAGGTGTTGGGTTCCTCCA<br>TGTGTCTTCAGGTGGCAAAGGA	60	NM_001244997	248
RANBP1	TCCACCGAGAATGCTGACGA<br>CCTTCCACTCTGGGAGGTCG	60	NM_001185070	166
SKA1	GCAGAGCTTCACTTTACCTCGC<br>TTGGCTGGTTCTTCAGGGTCA	386	XM_001926548	58
SLC51B	TTCAAGAGGTGACAGCCGTGT<br>TGCCTCTGTTCCTGGTTGGG	230	XM_003121716	58
SOD1	TCCATGTCCATCAGTTTGGA<br>CTGCCCAAGTCATCTGGTTT	250	NM_001190422	60
STEAP2	ATGTTGCCTACAGCCTCTGCT<br>GGATGGAGGTGACTGCCAGG	192	XM_003357423	60
TNF	CCAAGGACTCAGATCATCGT<br>GCTGGTTGTCTTTCAGCTTC	146	NM_214022	60
XDH	AGACCACAAGCAGGTCACCC<br>CGCTCCCCTTCAAAACGCAG	147	NM_001285974	60

Table S2 Fold change (FC) and *P*-value of all differentially expressed transcripts*

Gene Symbol	Gene Description	SINS high vs. SINS low FC	*P*-value	FDR
Upregulated				
FMO5	flavin containing monooxygenase 5	3.84	0.0494	0.482
CD207	CD207 molecule, langerin	3.75	0.0250	0.441
AKR1C1	aldo-keto reductase family 1, member C1	3.32	0.0197	0.429
CYP1A2	cytochrome P450, family 1, subfamily A, polypeptide 2	2.78	0.0291	0.453
ESM1	endothelial cell-specific molecule 1	2.70	0.0152	0.431
FGF14	fibroblast growth factor 14	2.47	0.0043	0.441
GYPA	glycophorin A (MNS blood group)	2.43	0.0383	0.466
LOC733635	aldo-keto reductase family 1 member C2-like	2.30	0.0080	0.419
EPB42	erythrocyte membrane protein band 4.2	2.25	0.0043	0.444
PHYHD1	phytanoyl-CoA dioxygenase domain containing 1	2.13	0.0067	0.422
LOC100736962	liver carboxylesterase	2.11	0.0247	0.440
HEMGN	hemogen	2.03	0.0123	0.429
SFTPA1	surfactant protein A1	2.03	0.0193	0.428
LOC100627616	calcium-activated potassium channel subunit alpha-1-like	2.02	0.0156	0.432
AKAP5	A kinase (PRKA) anchor protein 5	2.02	0.0003	0.555
ADGRG6	adhesion G protein-coupled receptor G6	1.97	0.0074	0.416
CRP	C-reactive protein, pentraxin-related	1.96	0.0253	0.444
LOC100738010	deoxyribonuclease gamma	1.93	0.0053	0.430
BCO2	beta-carotene oxygenase 2	1.91	0.0087	0.423
NRN1L	neuritin 1-like	1.90	0.0014	0.449
LOC100517145	complement C3	1.88	0.0299	0.456
TDO2	tryptophan 2,3-dioxygenase	1.88	0.0304	0.456
MPO	myeloperoxidase	1.86	0.0262	0.446
XDH	xanthine dehydrogenase	1.84	0.0226	0.436
CYP2E1	cytochrome P450, family 2, subfamily E, polypeptide 1	1.83	0.0388	0.467
AKR1C1	aldo-keto reductase family 1, member C1 (dihydrodiol dehydrogenase 1; 20-alpha (3-alpha)-hydroxysteroid dehydrogenase)	1.78	0.0051	0.430
AKR1C1	aldo-keto reductase family 1, member C1 (dihydrodiol dehydrogenase 1; 20-alpha (3-alpha)-hydroxysteroid dehydrogenase)	1.78	0.0051	0.432
LOC100512160	serine protease inhibitor Kazal-type 5	1.78	0.0237	0.438
LOC100513690	putative sodium-coupled neutral amino acid transporter 11	1.77	0.0007	0.630
DPYS	dihydropyrimidinase	1.76	0.0114	0.429
FNDC1	fibronectin type III domain containing 1	1.76	0.0307	0.457
MVK	mevalonate kinase	1.70	0.0006	0.650
CP	ceruloplasmin (ferroxidase)	1.68	0.0404	0.470
C4BPB	complement component 4 binding protein, beta	1.68	0.0149	0.429
CLEC2D	C-type lectin domain family 2, member D	1.68	0.0068	0.422
MAN1C1	mannosidase, alpha, class 1C, member 1	1.68	0.0073	0.419
RHAG	Rh-associated glycoprotein	1.67	0.0300	0.456
GRB7	growth factor receptor-bound protein 7	1.67	0.0198	0.429
CYP4F2	cytochrome P450, family 4, subfamily F, polypeptide 2	1.67	0.0223	0.436
WIPI1	WD repeat domain, phosphoinositide interacting 1	1.66	0.0054	0.431
ADHFE1	alcohol dehydrogenase, iron containing, 1	1.66	0.0219	0.436
VNN3	vanin 3	1.66	0.0173	0.429
KCNN2	potassium channel, calcium activated intermediate/small conductance subfamily N alpha, member 2	1.65	0.0299	0.455
ALAS2	5-aminolevulinate synthase 2	1.65	0.0073	0.419
KIF28P	kinesin family member 28, pseudogene	1.64	0.0103	0.422
INSIG1	insulin induced gene 1	1.64	0.0330	0.460
HSD11B1	hydroxysteroid (11-beta) dehydrogenase 1	1.63	0.0053	0.429
ACSM3	acyl-CoA synthetase medium-chain family member 3	1.63	0.0337	0.460
LOC100739121	uncharacterized LOC100739121	1.63	0.0115	0.427
IFIT3	interferon-induced protein with tetratricopeptide repeats 3	1.62	0.0414	0.473
MAT1A	methionine adenosyltransferase I, alpha	1.62	0.0252	0.443

ME3	malic enzyme 3, NADP(+)-dependent, mitochondrial	1.61	0.0284	0.452
01. Mrz	mitochondrial amidoxime reducing component 1	1.61	0.0469	0.478
MIR107	microRNA mir-107	1.59	0.0069	0.419
GCLC	glutamate-cysteine ligase, catalytic subunit	1.58	0.0364	0.465
APOF	apolipoprotein F	1.58	0.0219	0.436
HSD17B11	hydroxysteroid (17-beta) dehydrogenase 11	1.58	0.0137	0.433
GPR156	G protein-coupled receptor 156	1.57	0.0267	0.447
ZCCHC16	zinc finger, CCHC domain containing 16	1.57	0.0468	0.479
NNMT	nicotinamide N-methyltransferase	1.56	0.0479	0.480
TCN2	transcobalamin II	1.54	0.0002	0.682
PAIP2B	poly(A) binding protein interacting protein 2B	1.54	0.0070	0.416
SPIC	Spi-C transcription factor (Spi-1/PU.1 related)	1.54	0.0131	0.434
PPAT	phosphoribosyl pyrophosphate amidotransferase	1.53	0.0213	0.434
ACADS	acyl-CoA dehydrogenase, C-2 to C-3 short chain	1.51	0.0056	0.425
LOC100513372	probable 2-oxoglutarate dehydrogenase E1 component DHKTD1, mitochondrial	1.51	0.0153	0.431
MAGI3	membrane associated guanylate kinase, WW and PDZ domain containing 3	1.51	0.0168	0.436
HBB	hemoglobin, beta	1.50	0.0128	0.434
ETNK2	ethanolamine kinase 2	1.50	0.0425	0.476
LOC106507301	uncharacterized LOC106507301	1.50	0.0135	0.435
GPC4	glypican 4	1.49	0.0015	0.452
TLR4	toll-like receptor 4	1.49	0.0184	0.428
LOC100156705	melanoma-associated antigen 8-like	1.49	0.0100	0.421
AKR1C4	aldo-keto reductase family 1, member C4 (chlordecone reductase; 3-alpha hydroxysteroid dehydrogenase, type I; dihydrodiol dehydrogenase 4)	1.49	0.0347	0.464
PIK3C2G	phosphatidylinositol-4-phosphate 3-kinase, catalytic subunit type 2 gamma	1.49	0.0352	0.463
PRDM1	PR domain containing 1, with ZNF domain	1.48	0.0009	0.547
SLA-DMA	SLA-DM alpha chain	1.48	0.0459	0.479
SOAT2	sterol O-acyltransferase 2	1.48	0.0008	0.621
TEK	TEK tyrosine kinase, endothelial	1.48	0.0348	0.464
HERC3	HECT and RLD domain containing E3 ubiquitin protein ligase 3	1.47	0.0023	0.456
GNE	glucosamine (UDP-N-acetyl)-2-epimerase/N-acetylmannosamine kinase	1.47	0.0034	0.442
RNLS	renalase, FAD-dependent amine oxidase	1.47	0.0427	0.476
ATP2B2	ATPase, Ca++ transporting, plasma membrane 2	1.47	0.0373	0.466
SPTA1	spectrin, alpha, erythrocytic 1	1.46	0.0083	0.419
LOC100154752	olfactory receptor 6C3-like	1.46	0.0186	0.430
LOC100525819	FGGY carbohydrate kinase domain-containing protein	1.45	0.0168	0.435
SEMA6A	sema domain, transmembrane domain (TM), and cytoplasmic domain, (semaphorin) 6A	1.45	0.0176	0.429
KCNT2	potassium channel, sodium activated subfamily T, member 2	1.45	0.0055	0.427
HIP1R	huntingtin interacting protein 1 related	1.45	0.0049	0.437
MMP19	matrix metallopeptidase 19	1.45	0.0038	0.440
DLG2	discs, large homolog 2 (Drosophila)	1.45	0.0168	0.435
MCTP1	multiple C2 domains, transmembrane 1	1.45	0.0045	0.438
LOC396634	receptor-type tyrosine-protein phosphatase C-like	1.45	0.0490	0.481
SCIMP	SLP adaptor and CSK interacting membrane protein	1.45	0.0013	0.476
MAB21L3	mab-21-like 3 (C. elegans)	1.44	0.0029	0.428
C4A	complement component 4A (Rodgers blood group)	1.44	0.0103	0.423
TRPC5	transient receptor potential cation channel, subfamily C, member 5	1.44	0.0166	0.436
LOC102160410	uncharacterized LOC102160410	1.44	0.0163	0.437
LOC100520032	myeloid-associated differentiation marker-like	1.44	0.0316	0.459
TAT	tyrosine aminotransferase	1.44	0.0490	0.481
ATP6V1G2	ATPase, H+ transporting, lysosomal 13kDa, V1 subunit G2	1.44	0.0019	0.452
LOC100518644	glycine N-acyltransferase-like	1.44	0.0447	0.478

FMO1	flavin containing monooxygenase 1	1.44	0.0298	0.456
EFNA1	ephrin-A1	1.44	0.0352	0.462
LOC100511504	maestro heat-like repeat-containing protein family member 1	1.43	0.0034	0.441
LOC100736871	myeloid-associated differentiation marker-like	1.43	0.0034	0.439
CYP39A1	cytochrome P450, family 39, subfamily A, polypeptide 1	1.43	0.0432	0.476
LOC100523985	cytoplasmic dynein 1 intermediate chain 1	1.43	0.0468	0.479
LOC100737684	estrogen receptor-like	1.43	0.0493	0.482
MATN2	matrilin 2	1.43	0.0079	0.420
CPLX4	complexin 4	1.43	0.0177	0.428
DEPTOR	DEP domain containing MTOR-interacting protein	1.42	0.0116	0.426
LOC100156240	CDK5 regulatory subunit-associated protein 2	1.42	0.0367	0.465
RSC1A1	regulatory solute carrier protein, family 1, member 1	1.42	0.0356	0.463
NFKBIZ	nuclear factor of kappa light polypeptide gene enhancer in B-cells inhibitor, zeta	1.42	0.0066	0.427
LOC100621650	protein furry homolog	1.42	0.0373	0.466
YPEL5	yippee-like 5	1.42	0.0080	0.419
LOC100739748	glutaryl-CoA dehydrogenase, mitochondrial-like	1.42	0.0405	0.470
C5	complement component 5	1.42	0.0144	0.430
ALDH6A1	aldehyde dehydrogenase 6 family, member A1	1.42	0.0278	0.448
ZNF366	zinc finger protein 366	1.42	0.0340	0.462
LOC100737965	runt-related transcription factor 2	1.42	0.0405	0.470
ADCK3	aarF domain containing kinase 3	1.41	0.0289	0.454
VSIG4	V-set and immunoglobulin domain containing 4	1.41	0.0227	0.436
SMARCA2	SWI/SNF related, matrix associated, actin dependent regulator of chromatin, subfamily a, member 2	1.41	0.0093	0.425
LOC100739233	nidogen-1-like	1.41	0.0131	0.433
LRG1	leucine-rich alpha-2-glycoprotein 1	1.41	0.0110	0.428
HAO2	hydroxyacid oxidase 2 (long chain)	1.41	0.0151	0.429
FIGF	c-fos induced growth factor (vascular endothelial growth factor D)	1.41	0.0242	0.440
RIBC1	RIB43A domain with coiled-coils 1	1.40	0.0022	0.450
UROC1	urocanate hydratase 1	1.40	0.0460	0.479
DCAF11	DDB1 and CUL4 associated factor 11	1.40	0.0137	0.435
TIAM1	T-cell lymphoma invasion and metastasis 1	1.40	0.0067	0.423
BHLHB9	basic helix-loop-helix domain containing, class B, 9	1.40	0.0188	0.430
RBP7	retinol binding protein 7, cellular	1.40	0.0149	0.429
FAM198B	family with sequence similarity 198, member B	1.40	0.0367	0.466
CTNS	cystinosin, lysosomal cystine transporter	1.40	0.0312	0.457
TNFSF14	tumor necrosis factor (ligand) superfamily, member 14	1.39	0.0009	0.598
IVD	isovaleryl-CoA dehydrogenase	1.39	0.0136	0.436
C1H6orf211	uncharacterized protein C6orf211 homolog	1.39	0.0434	0.476
LOC100737517	solute carrier family 40 member 1	1.39	0.0454	0.478
AQP11	aquaporin 11	1.39	0.0066	0.424
LOC100738724	24-hydroxycholesterol 7-alpha-hydroxylase-like	1.39	0.0252	0.443
TMEM120B	transmembrane protein 120B	1.39	0.0469	0.478
LOC100512076	solute carrier family 40 member 1	1.39	0.0467	0.480
LOC100737638	nesprin-1	1.39	0.0174	0.429
WBP1L	WW domain binding protein 1-like	1.39	0.0372	0.466
PINK1	PTEN induced putative kinase 1	1.39	0.0307	0.457
KEL	Kell blood group, metallo-endopeptidase	1.39	0.0353	0.463
CTSS	cathepsin S	1.39	0.0307	0.457
LOC100737308	D-beta-hydroxybutyrate dehydrogenase, mitochondrial-like	1.39	0.0445	0.478
CHDH	choline dehydrogenase	1.39	0.0471	0.479
ALDH5A1	aldehyde dehydrogenase 5 family, member A1	1.39	0.0011	0.509
LOC100513779	solute carrier family 22 member 9-like	1.38	0.0386	0.467
CCDC69	coiled-coil domain containing 69	1.38	0.0396	0.469
CNTN4	contactin 4	1.38	0.0246	0.440
CSF1R	colony stimulating factor 1 receptor	1.38	0.0165	0.433
SUSD6	sushi domain containing 6	1.38	0.0173	0.429
LOC100517129	nuclear body protein SP140-like protein	1.38	0.0217	0.436

PLA1A	phospholipase A1 member A	1.38	0.0444	0.477
LOC100515273	putative 2-oxo-4-hydroxy-4-carboxy-5-ureidoimidazoline decarboxylase	1.38	0.0081	0.419
PRC1	protein regulator of cytokinesis 1	1.38	0.0164	0.435
PTPRC	receptor-type tyrosine-protein phosphatase C	1.37	0.0442	0.478
SATB1	SATB homeobox 1	1.37	0.0259	0.448
LOC100520861	ATP-binding cassette sub-family A member 6	1.37	0.0084	0.420
AUH	AU RNA binding protein/enoyl-CoA hydratase	1.37	0.0424	0.476
NAGA	N-acetylgalactosaminidase, alpha-	1.37	0.0027	0.426
MAP2K6	mitogen-activated protein kinase kinase 6	1.37	0.0484	0.481
LOC100519209	BDNF/NT-3 growth factors receptor	1.36	0.0116	0.424
LOC100155953	serpin A11	1.36	0.0092	0.426
LOC102160066	uncharacterized LOC102160066	1.36	0.0419	0.475
FAM213A	family with sequence similarity 213, member A	1.36	0.0148	0.429
CD200R1	CD200 receptor 1	1.36	0.0380	0.466
CXCL11	chemokine (C-X-C motif) ligand 11	1.36	0.0487	0.481
GCDH	glutaryl-CoA dehydrogenase	1.36	0.0227	0.435
KAT6B	K(lysine) acetyltransferase 6B	1.36	0.0043	0.442
CUX2	cut-like homeobox 2	1.36	0.0422	0.476
LRRC8C	leucine rich repeat containing 8 family, member C	1.35	0.0246	0.441
CLCN6	chloride channel, voltage-sensitive 6	1.35	0.0073	0.419
RENBP	renin binding protein	1.35	0.0036	0.443
CCDC122	coiled-coil domain containing 122	1.35	0.0304	0.456
LOC100156587	olfactory receptor 4M1	1.35	0.0141	0.432
SDC2	syndecan 2	1.35	0.0028	0.431
CACNA2D2	calcium channel, voltage-dependent, alpha 2/delta subunit 2	1.34	0.0046	0.434
DGAT2	diacylglycerol O-acyltransferase 2	1.34	0.0423	0.476
DBT	dihydrolipoamide branched chain transacylase E2	1.34	0.0056	0.426
AGMO	alkylglycerol monooxygenase	1.34	0.0122	0.431
HSF5	heat shock transcription factor family member 5	1.34	0.0372	0.466
PPP1R18	protein phosphatase 1, regulatory subunit 18	1.34	0.0172	0.431
LOC100737768	hemoglobin subunit alpha	1.34	0.0341	0.462
APBB3	amyloid beta (A4) precursor protein-binding, family B, member 3	1.34	0.0463	0.480
LOC100520083	molybdenum cofactor sulfurase-like	1.34	0.0359	0.464
IL7	interleukin 7	1.33	0.0211	0.435
LOC100521751	nephrocystin-3-like	1.33	0.0200	0.430
HPS3	Hermansky-Pudlak syndrome 3	1.33	0.0314	0.458
FGD2	FYVE, RhoGEF and PH domain containing 2	1.33	0.0397	0.469
CXCR4	chemokine (C-X-C motif) receptor 4	1.33	0.0383	0.466
FOXN3	forkhead box N3	1.33	0.0153	0.432
ZNF655	zinc finger protein 655	1.33	0.0025	0.435
LOC100519394	methyl-CpG-binding domain protein 5	1.33	0.0154	0.431
TSPAN3	tetraspanin 3	1.32	0.0172	0.431
TMEM204	transmembrane protein 204	1.32	0.0154	0.432
LOC100520241	olfactory receptor 10J1-like	1.32	0.0385	0.466
LOC100520241	olfactory receptor 10J1-like	1.32	0.0385	0.467
LOC100518411	anoctamin-1	1.32	0.0047	0.434
CTSK	cathepsin K	1.32	0.0308	0.457
PCK2	phosphoenolpyruvate carboxykinase 2 (mitochondrial)	1.32	0.0418	0.475
MIR144	microRNA mir-144	1.32	0.0315	0.459
LOC100621998	stannin	1.32	0.0109	0.430
LOC100739280	pro-neuregulin-1, membrane-bound isoform-like	1.32	0.0267	0.448
MAP10	microtubule-associated protein 10	1.32	0.0170	0.433
GLRX5	glutaredoxin 5	1.32	0.0012	0.473
ADGRL2	adhesion G protein-coupled receptor L2	1.32	0.0322	0.458
ARHGAP30	Rho GTPase activating protein 30	1.32	0.0177	0.428
ACAA1	acetyl-CoA acyltransferase 1	1.31	0.0039	0.440
TRERF1	transcriptional regulating factor 1	1.31	0.0444	0.477
LOC100738248	endonuclease/exonuclease/phosphatase family domain-containing protein 1-like	1.31	0.0031	0.433

LOC100524975	olfactory receptor 10H1	1.31	0.0337	0.460
IFN-ALPHA-16	interferon-alpha-16	1.31	0.0062	0.424
HSD17B10	hydroxysteroid (17-beta) dehydrogenase 10	1.31	0.0219	0.436
SCARA5	scavenger receptor class A, member 5	1.31	0.0243	0.441
LPIN3	lipin 3	1.31	0.0136	0.436
CLK1	CDC-like kinase 1	1.31	0.0310	0.456
Downregulated				
CSPG4	chondroitin sulfate proteoglycan 4	-1.31	0.0278	0.448
RPL12	ribosomal protein L12	-1.31	0.0101	0.423
ICMT	isoprenylcysteine carboxyl methyltransferase	-1.31	0.0051	0.430
RCC2	regulator of chromosome condensation 2	-1.31	0.0164	0.435
SFXN4	sideroflexin 4	-1.31	0.0146	0.430
LOC100737729	polyribonucleotide nucleotidyltransferase 1, mitochondrial-like	-1.31	0.0459	0.479
PERP	PERP, TP53 apoptosis effector	-1.31	0.0262	0.447
FAM20B	family with sequence similarity 20, member B	-1.31	0.0151	0.430
ORMDL1	ORMDL sphingolipid biosynthesis regulator 1	-1.31	0.0391	0.469
NDUFS6	NADH dehydrogenase (ubiquinone) Fe-S protein 6, 13kDa (NADH-coenzyme Q reductase)	-1.31	0.0014	0.470
MRPL10	mitochondrial ribosomal protein L10	-1.31	0.0070	0.420
LOC100623012	cofilin-1 pseudogene	-1.31	0.0396	0.469
HNRNPA1	heterogeneous nuclear ribonucleoprotein A1	-1.31	0.0338	0.461
LSM6	LSM6 homolog, U6 small nuclear RNA and mRNA degradation associated	-1.31	0.0170	0.432
RPS6KL1	ribosomal protein S6 kinase-like 1	-1.31	0.0329	0.459
PGAM1	phosphoglycerate mutase 1 (brain)	-1.31	0.0466	0.480
VMP1	vacuole membrane protein 1	-1.31	0.0205	0.434
MCU	mitochondrial calcium uniporter	-1.31	0.0226	0.437
USP10	ubiquitin specific peptidase 10	-1.31	0.0227	0.435
URM1 // URM1	ubiquitin related modifier 1 // ubiquitin related modifier 1	-1.31	0.0272	0.448
ANAPC5	anaphase promoting complex subunit 5	-1.31	0.0489	0.481
GNAI1	guanine nucleotide binding protein (G protein), alpha inhibiting activity polypeptide 1	-1.31	0.0115	0.430
MRPS14	mitochondrial ribosomal protein S14	-1.31	0.0137	0.433
RAD51C	RAD51 paralog C	-1.31	0.0384	0.466
DYNC1LI1	dynein, cytoplasmic 1, light intermediate chain 1	-1.31	0.0075	0.418
SSB	Sjogren syndrome antigen B (autoantigen La)	-1.31	0.0096	0.421
COPS7A	COP9 signalosome subunit 7A	-1.31	0.0318	0.459
RPL31	ribosomal protein L31	-1.31	0.0019	0.456
PRNP	prion protein	-1.32	0.0393	0.470
UBA2	ubiquitin-like modifier activating enzyme 2	-1.32	0.0201	0.431
MRPL21	mitochondrial ribosomal protein L21	-1.32	0.0042	0.440
PTBP1	polypyrimidine tract binding protein 1	-1.32	0.0115	0.430
MRPL11	mitochondrial ribosomal protein L11	-1.32	0.0353	0.462
CRYM	crystallin, mu	-1.32	0.0261	0.447
RQCD1	RCD1 required for cell differentiation1 homolog (S. pombe)	-1.32	0.0494	0.482
MLST8	MTOR associated protein, LST8 homolog	-1.32	0.0391	0.469
HSPA9	heat shock 70kDa protein 9 (mortalin)	-1.32	0.0041	0.444
G3BP1	GTPase activating protein (SH3 domain) binding protein 1	-1.32	0.0032	0.435
UTP15	UTP15, U3 small nucleolar ribonucleoprotein, homolog (S. cerevisiae)	-1.32	0.0016	0.457
METAP1	methionyl aminopeptidase 1	-1.32	0.0091	0.425
TAGLN2	transgelin 2	-1.32	0.0442	0.478
HNRNPD	heterogeneous nuclear ribonucleoprotein D (AU-rich element RNA binding protein 1, 37kDa)	-1.32	0.0039	0.437
EIF2S1	eukaryotic translation initiation factor 2, subunit 1 alpha, 35kDa	-1.32	0.0168	0.436
CCNC	cyclin C	-1.32	0.0067	0.422

ZBTB10	zinc finger and BTB domain containing 10	-1.32	0.0327	0.459
ZNF280B	zinc finger protein 280B	-1.32	0.0196	0.429
LOC100525232	cytochrome c oxidase assembly protein COX16 homolog, mitochondrial	-1.32	0.0094	0.422
LOC100520848	prickle-like protein 4	-1.33	0.0036	0.443
LOC100738600	prickle-like protein 4	-1.33	0.0036	0.445
ADSL	adenylosuccinate lyase	-1.33	0.0259	0.447
MYBBP1A	MYB binding protein (P160) 1a	-1.33	0.0475	0.480
LOC100739335	ruvB-like 1	-1.33	0.0051	0.437
THOP1	thimet oligopeptidase 1	-1.33	0.0031	0.429
E2F4	E2F transcription factor 4, p107/p130-binding	-1.33	0.0316	0.459
UCHL5	ubiquitin carboxyl-terminal hydrolase L5	-1.33	0.0115	0.430
CDK20	cyclin-dependent kinase 20	-1.33	0.0139	0.432
GSTA4	glutathione S-transferase alpha 4	-1.33	0.0411	0.472
BPNT1	3(2), 5-bisphosphate nucleotidase 1	-1.33	0.0012	0.462
RRP12	ribosomal RNA processing 12 homolog	-1.33	0.0323	0.458
CPLX3	complexin 3	-1.33	0.0107	0.428
SLC9B1	solute carrier family 9, subfamily B (NHA1, cation proton antiporter 1), member 1	-1.33	0.0378	0.467
MRS2	MRS2 magnesium transporter	-1.33	0.0472	0.479
PNO1	partner of NOB1 homolog	-1.33	0.0082	0.418
ETF1	eukaryotic translation termination factor 1	-1.33	0.0068	0.423
QRSL1	glutaminyl-tRNA synthase (glutamine-hydrolyzing)-like 1	-1.33	0.0043	0.446
POLE3	polymerase (DNA directed), epsilon 3, accessory subunit	-1.33	0.0154	0.431
LARS	leucyl-tRNA synthetase	-1.33	0.0422	0.476
CEP78	centrosomal protein 78kDa	-1.34	0.0223	0.437
MRPS35	mitochondrial ribosomal protein S35	-1.34	0.0019	0.454
C4H8orf59	chromosome 4 open reading frame, human C8orf59	-1.34	0.0103	0.422
PRRC2A	proline-rich coiled-coil 2A	-1.34	0.0063	0.425
TMEM33	transmembrane protein 33	-1.34	0.0116	0.425
SLC25A5	solute carrier family 25 (mitochondrial carrier; adenine nucleotide translocator), member 5	-1.34	0.0179	0.429
HSF2	heat shock transcription factor 2	-1.34	0.0116	0.424
TXNDC9	thioredoxin domain containing 9	-1.34	0.0053	0.429
MMGT1	membrane magnesium transporter 1	-1.34	0.0107	0.428
YWHAQ	tyrosine 3-monooxygenase/tryptophan 5-monooxygenase activation protein, theta	-1.34	0.0333	0.459
EIF2S2	eukaryotic translation initiation factor 2, subunit 2 beta, 38kDa	-1.34	0.0152	0.431
EIF2S2	eukaryotic translation initiation factor 2, subunit 2 beta, 38kDa	-1.34	0.0152	0.431
CHCHD3	coiled-coil-helix-coiled-coil-helix domain containing 3	-1.34	0.0024	0.447
DDX21	DEAD (Asp-Glu-Ala-Asp) box helicase 21	-1.34	0.0217	0.436
RPUSD3	RNA pseudouridylate synthase domain containing 3	-1.34	0.0289	0.454
EXOSC2	exosome component 2	-1.34	0.0002	0.737
TRMT5	tRNA methyltransferase 5	-1.34	0.0092	0.425
LOC100738939	nuclear envelope integral membrane protein 1	-1.34	0.0466	0.479
LOC100738939	nuclear envelope integral membrane protein 1	-1.34	0.0466	0.480
MITD1	MIT, microtubule interacting and transport, domain containing 1	-1.34	0.0059	0.423
PTCD3	pentatricopeptide repeat domain 3	-1.34	0.0013	0.469
IL27RA	interleukin 27 receptor, alpha	-1.34	0.0311	0.457
ERH	enhancer of rudimentary homolog (Drosophila)	-1.34	0.0282	0.449
LOC102161054	olfactory receptor 7G3-like	-1.34	0.0080	0.419
COPS3	COP9 signalosome subunit 3	-1.34	0.0127	0.431
YWHAZ	tyrosine 3-monooxygenase/tryptophan 5-monooxygenase activation protein, zeta	-1.34	0.0428	0.476
LOC100525076	solute carrier family 35 member B1	-1.34	0.0476	0.480
PRKAR2A	protein kinase, cAMP-dependent, regulatory, type II, alpha	-1.34	0.0077	0.421
MRI1	methylthioribose-1-phosphate isomerase 1	-1.35	0.0138	0.430
YKT6	YKT6 v-SNARE homolog (S. cerevisiae)	-1.35	0.0433	0.476

KCNH6	potassium channel, voltage gated eag related subfamily H, member 6	-1.35	0.0012	0.455
METTL16	methyltransferase like 16	-1.35	0.0075	0.419
SNAPC3	small nuclear RNA activating complex, polypeptide 3, 50kDa	-1.35	0.0011	0.490
ANP32A	acidic (leucine-rich) nuclear phosphoprotein 32 family, member A	-1.35	0.0239	0.439
DTD2	D-tyrosyl-tRNA deacylase 2 (putative)	-1.35	0.0205	0.433
CCNJ	cyclin J	-1.35	0.0013	0.473
BID	BH3 interacting domain death agonist	-1.35	0.0040	0.439
HSPA14	heat shock 70kDa protein 14	-1.35	0.0075	0.418
MAGED1	melanoma antigen family D, 1	-1.35	0.0350	0.462
FAM167B	family with sequence similarity 167, member B	-1.35	0.0454	0.478
MAMLD1	mastermind-like domain containing 1	-1.35	0.0486	0.481
CCNYL1	cyclin Y-like 1	-1.36	0.0089	0.428
IBTK	inhibitor of Bruton agammaglobulinemia tyrosine kinase	-1.36	0.0382	0.466
TRMT2A	tRNA methyltransferase 2 homolog A (S. cerevisiae)	-1.36	0.0169	0.434
MMP7	matrix metallopeptidase 7	-1.36	0.0270	0.449
ANP32A	acidic (leucine-rich) nuclear phosphoprotein 32 family, member A	-1.36	0.0139	0.431
NDUFC1	NADH dehydrogenase (ubiquinone) 1, subcomplex unknown, 1, 6kDa	-1.36	0.0294	0.455
EXOSC4	exosome component 4	-1.36	0.0124	0.429
TAF11	TAF11 RNA polymerase II, TATA box binding protein (TBP)-associated factor, 28kDa	-1.36	0.0055	0.429
YBX3	Y box binding protein 3	-1.36	0.0399	0.469
LOC102161719	uncharacterized LOC102161719	-1.36	0.0057	0.421
SMIM11A	small integral membrane protein 11A	-1.36	0.0096	0.421
DHX35	DEAH (Asp-Glu-Ala-His) box polypeptide 35	-1.37	0.0056	0.424
TKFC	triokinase/FMN cyclase	-1.37	0.0331	0.459
GEMIN6	gem (nuclear organelle) associated protein 6	-1.37	0.0093	0.424
HAUS6	HAUS augmin-like complex, subunit 6	-1.37	0.0193	0.428
NDUFA8	NADH dehydrogenase (ubiquinone) 1 alpha subcomplex, 8, 19kDa	-1.37	0.0215	0.436
LOC100738751	F-box only protein 6-like	-1.37	0.0066	0.426
PRPS1	phosphoribosyl pyrophosphate synthetase 1	-1.37	0.0235	0.437
MRPL53	mitochondrial ribosomal protein L53	-1.37	0.0170	0.433
UTP20	UTP20, small subunit (SSU) processome component, homolog (yeast)	-1.37	0.0095	0.422
GNL3	guanine nucleotide binding protein-like 3 (nucleolar)	-1.37	0.0109	0.429
AGPAT1	1-acylglycerol-3-phosphate O-acyltransferase 1	-1.37	0.0078	0.417
PSME3	proteasome activator subunit 3	-1.37	0.0019	0.453
LOC100739849	28S ribosomal protein S16, mitochondrial	-1.37	0.0047	0.434
NAA20	N(alpha)-acetyltransferase 20, NatB catalytic subunit	-1.37	0.0070	0.416
EIF4A1	eukaryotic translation initiation factor 4A1	-1.38	0.0104	0.422
LOC100737594	exportin-5-like	-1.38	0.0186	0.430
SNRPD3	small nuclear ribonucleoprotein D3 polypeptide 18kDa	-1.38	0.0157	0.432
GALT	galactose-1-phosphate uridylyltransferase	-1.38	0.0437	0.477
SLC25A22	solute carrier family 25 (mitochondrial carrier: glutamate), member 22	-1.38	0.0476	0.480
LOC100152843	forkhead box protein Q1	-1.38	0.0006	0.594
SUZ12	SUZ12 polycomb repressive complex 2 subunit	-1.38	0.0101	0.423
DAP	death-associated protein	-1.38	0.0176	0.429
NPM1	nucleophosmin (nucleolar phosphoprotein B23, numatrin)	-1.38	0.0004	0.579
CCT2	chaperonin containing TCP1, subunit 2 (beta)	-1.38	0.0025	0.451
PDCD11	programmed cell death 11	-1.38	0.0119	0.429
LOC100524598	cytochrome c-type heme lyase	-1.38	0.0409	0.472
LOC100155823	olfactory receptor 4F3/4F16/4F29-like	-1.38	0.0403	0.470
NUTF2	nuclear transport factor 2	-1.38	0.0121	0.429
PPIL1	peptidylprolyl isomerase (cyclophilin)-like 1	-1.38	0.0208	0.433
CDK2	cyclin-dependent kinase 2	-1.38	0.0076	0.419

SCAMP5	secretory carrier membrane protein 5	-1.39	0.0078	0.417
SRSF7	serine/arginine-rich splicing factor 7	-1.39	0.0154	0.431
LOC100157467	DNA polymerase alpha catalytic subunit	-1.39	0.0415	0.474
CFL2	cofilin 2 (muscle)	-1.39	0.0035	0.440
MAGOH	mago homolog, exon junction complex core component	-1.39	0.0055	0.430
MRPL13	mitochondrial ribosomal protein L13	-1.39	0.0041	0.444
NUP160	nucleoporin 160kDa	-1.39	0.0051	0.428
FHL3	four and a half LIM domains 3	-1.39	0.0005	0.582
CCT7	chaperonin containing TCP1, subunit 7 (eta)	-1.39	0.0195	0.429
CCDC91	coiled-coil domain containing 91	-1.39	0.0307	0.456
FAM98A	family with sequence similarity 98, member A	-1.39	0.0354	0.462
TMEM167A	transmembrane protein 167A	-1.39	0.0194	0.427
UTP15	UTP15, U3 small nucleolar ribonucleoprotein, homolog (S. cerevisiae)	-1.39	0.0237	0.438
DOCK5	dedicator of cytokinesis 5	-1.39	0.0378	0.467
IDH3A	isocitrate dehydrogenase 3 (NAD+) alpha	-1.39	0.0081	0.418
INTS7	integrator complex subunit 7	-1.39	0.0169	0.435
LOC100737174	nucleolin-like	-1.39	0.0046	0.433
LOC100155138	tubulin alpha-3 chain	-1.40	0.0101	0.422
LOC100739768	beta-parvin	-1.40	0.0275	0.448
PPP1R14B	protein phosphatase 1, regulatory (inhibitor) subunit 14B	-1.40	0.0320	0.459
PPID	peptidylprolyl isomerase D	-1.40	0.0459	0.479
NANP	N-acetylneuraminic acid phosphatase	-1.40	0.0250	0.441
LOC100737210	nucleolin	-1.40	0.0096	0.421
LOC102167832	uncharacterized LOC102167832	-1.40	0.0060	0.423
KPNB1	karyopherin (importin) beta 1	-1.40	0.0212	0.435
RAB23	RAB23, member RAS oncogene family	-1.40	0.0220	0.437
PLEKHA8	pleckstrin homology domain containing, family A (phosphoinositide binding specific) member 8	-1.40	0.0297	0.456
APEX1	APEX nuclease (multifunctional DNA repair enzyme) 1	-1.40	0.0218	0.436
PRELID3B	PRELI domain containing 3B	-1.40	0.0051	0.434
NOP56	NOP56 ribonucleoprotein	-1.40	0.0069	0.422
LOC102163346	malate dehydrogenase, cytoplasmic	-1.40	0.0224	0.436
DCLRE1B	DNA cross-link repair 1B	-1.40	0.0189	0.431
AAAS	achalasia, adrenocortical insufficiency, alacrimia	-1.40	0.0162	0.437
SRPK1	SRSF protein kinase 1	-1.40	0.0002	0.757
APEX2	APEX nuclease (apurinic/apyrimidinic endonuclease) 2	-1.40	0.0265	0.446
POLR3D	polymerase (RNA) III (DNA directed) polypeptide D, 44kDa	-1.41	0.0176	0.429
LSM3	LSM3 homolog, U6 small nuclear RNA and mRNA degradation associated	-1.41	0.0105	0.422
MRPS30	mitochondrial ribosomal protein S30	-1.41	0.0183	0.428
LOC100523894	DNA-directed RNA polymerase III subunit RPC5	-1.41	0.0068	0.422
TWF1	twinfilin actin binding protein 1	-1.41	0.0182	0.429
CWC15	CWC15 spliceosome-associated protein	-1.41	0.0060	0.423
PHF6	PHD finger protein 6	-1.41	0.0090	0.424
TIMM8A	translocase of inner mitochondrial membrane 8 homolog A (yeast)	-1.41	0.0285	0.451
SUV39H2	suppressor of variegation 3-9 homolog 2 (Drosophila)	-1.41	0.0312	0.457
ILF2	interleukin enhancer binding factor 2	-1.42	0.0074	0.418
NOLC1	nucleolar and coiled-body phosphoprotein 1	-1.42	0.0053	0.430
ARF6	ADP-ribosylation factor 6	-1.42	0.0070	0.417
LOC100519524	origin recognition complex subunit 3	-1.42	0.0422	0.476
TRIM37	tripartite motif containing 37	-1.42	0.0014	0.462
CEP57L1	centrosomal protein 57kDa-like 1	-1.42	0.0004	0.586
PGC	progastricsin (pepsinogen C)	-1.42	0.0403	0.470
LOC100519729	tax1-binding protein 3-like	-1.43	0.0070	0.419
ZDHHC21	zinc finger, DHHC-type containing 21	-1.43	0.0327	0.459
AATF	apoptosis antagonizing transcription factor	-1.43	0.0305	0.456
UTP6	UTP6, small subunit (SSU) processome component, homolog (yeast)	-1.43	0.0257	0.447

AVEN	apoptosis, caspase activation inhibitor	-1.43	0.0383	0.466
HSPD1	heat shock 60kDa protein 1 (chaperonin)	-1.43	0.0055	0.431
FRRS1	ferric-chelate reductase 1	-1.43	0.0441	0.478
PIGU	phosphatidylinositol glycan anchor biosynthesis, class U	-1.43	0.0434	0.475
CASP2	caspase 2, apoptosis-related cysteine peptidase	-1.43	0.0131	0.433
ABHD6	abhydrolase domain containing 6	-1.43	0.0446	0.478
LOC100517015	cAMP-dependent protein kinase inhibitor beta-like	-1.43	0.0187	0.430
TUBA8	tubulin, alpha 8	-1.43	0.0224	0.436
PAK1IP1	PAK1 interacting protein 1	-1.43	0.0239	0.440
SMIM8	small integral membrane protein 8	-1.44	0.0038	0.437
WDR77	WD repeat domain 77	-1.44	0.0202	0.432
HSPA8	heat shock 70kDa protein 8	-1.44	0.0389	0.468
BIVM // BIVM	basic, immunoglobulin-like variable motif containing // basic, immunoglobulin-like variable motif containing	-1.44	0.0200	0.430
FASTKD2	FAST kinase domains 2	-1.44	0.0142	0.431
POLD3	polymerase (DNA-directed), delta 3, accessory subunit	-1.44	0.0141	0.430
RBPMS2	RNA binding protein with multiple splicing 2	-1.44	0.0299	0.456
MRE11A	MRE11 homolog A, double strand break repair nuclease	-1.44	0.0176	0.429
FARSA	phenylalanyl-tRNA synthetase, alpha subunit	-1.44	0.0359	0.464
CCT4	chaperonin containing TCP1, subunit 4 (delta)	-1.44	0.0224	0.436
NAP1L4	nucleosome assembly protein 1-like 4	-1.44	0.0057	0.421
FKBP3	FK506 binding protein 3, 25kDa	-1.44	0.0295	0.455
SGMS2	sphingomyelin synthase 2	-1.44	0.0215	0.435
DNAJB1	DnaJ (Hsp40) homolog, subfamily B, member 1	-1.44	0.0340	0.463
DYNLL1	dynein, light chain, LC8-type 1	-1.45	0.0166	0.436
KRT8	keratin 8, type II	-1.45	0.0429	0.476
MIF	macrophage migration inhibitory factor (glycosylation-inhibiting factor)	-1.45	0.0118	0.426
RAD54L	RAD54-like (S. cerevisiae)	-1.45	0.0377	0.466
PIGY	phosphatidylinositol glycan anchor biosynthesis, class Y	-1.45	0.0160	0.434
HVCN1	hydrogen voltage gated channel 1	-1.45	0.0299	0.456
ATP5G1	ATP synthase, H+ transporting, mitochondrial Fo complex, subunit C1 (subunit 9)	-1.45	0.0331	0.459
CCDC34	coiled-coil domain containing 34	-1.45	0.0305	0.456
TIMELESS	timeless circadian clock	-1.45	0.0397	0.469
ERI2	ERI1 exoribonuclease family member 2	-1.45	0.0247	0.441
EIF4A3	eukaryotic translation initiation factor 4A3	-1.46	0.0026	0.434
UMPS	uridine monophosphate synthetase	-1.46	0.0063	0.427
SMN1	survival of motor neuron 1, telomeric	-1.46	0.0099	0.421
LOC100153768	acidic leucine-rich nuclear phosphoprotein 32 family member E	-1.46	0.0396	0.469
LOC100515066	U6 snRNA-associated Sm-like protein LSm8	-1.46	0.0038	0.441
LOC100515066	U6 snRNA-associated Sm-like protein LSm8	-1.46	0.0038	0.443
LRRC40	leucine rich repeat containing 40	-1.47	0.0120	0.429
DDX18	DEAD (Asp-Glu-Ala-Asp) box polypeptide 18	-1.47	0.0102	0.423
CACYBP	calcyclin binding protein	-1.47	0.0063	0.426
RAN	RAN, member RAS oncogene family	-1.47	0.0165	0.434
DPCD	deleted in primary ciliary dyskinesia homolog (mouse)	-1.47	0.0004	0.597
DENR	density-regulated protein	-1.47	0.0127	0.432
DKC1	dyskeratosis congenita 1, dyskerin	-1.47	0.0065	0.430
ATF3	activating transcription factor 3	-1.48	0.0129	0.435
TIMM9	translocase of inner mitochondrial membrane 9 homolog (yeast)	-1.48	0.0060	0.424
LTA4H	leukotriene A4 hydrolase	-1.49	0.0002	0.865
TTC27	tetratricopeptide repeat domain 27	-1.49	0.0086	0.421
PDCD5	programmed cell death 5	-1.49	0.0145	0.430
CCT3	chaperonin containing TCP1, subunit 3 (gamma)	-1.49	0.0026	0.428
NUP155	nucleoporin 155kDa	-1.50	0.0082	0.416
CHORDC1	cysteine and histidine-rich domain (CHORD) containing 1	-1.50	0.0078	0.417
TAMM41	TAM41, mitochondrial translocator assembly and maintenance protein, homolog (S. cerevisiae)	-1.50	0.0347	0.465

RPF2	ribosome production factor 2 homolog	-1.50	0.0099	0.421
POLA2	polymerase (DNA directed), alpha 2, accessory subunit	-1.50	0.0365	0.466
PSMA5	proteasome subunit alpha 5	-1.50	0.0046	0.433
VDAC2	voltage-dependent anion channel 2	-1.50	0.0092	0.425
LOC100157391	mitochondrial import inner membrane translocase subunit Tim23	-1.50	0.0058	0.422
FAM136A	family with sequence similarity 136, member A	-1.50	0.0071	0.416
HSPA4	heat shock 70kDa protein 4	-1.51	0.0032	0.436
CENPV	centromere protein V	-1.51	0.0000	1.203
ACOT4	acyl-CoA thioesterase 4	-1.51	0.0374	0.466
NUP43	nucleoporin 43kDa	-1.51	0.0044	0.438
LOC100519773	proliferation-associated protein 2G4	-1.51	0.0052	0.428
TUBG1	tubulin, gamma 1	-1.51	0.0037	0.442
PLN	phospholamban	-1.52	0.0086	0.421
HIGD1A	HIG1 hypoxia inducible domain family, member 1A	-1.52	0.0159	0.434
SRSF3	serine/arginine-rich splicing factor 3	-1.52	0.0073	0.419
LOC102158249	uncharacterized protein KIAA1841	-1.52	0.0018	0.455
PRMT1	protein arginine methyltransferase 1	-1.52	0.0140	0.432
CDK2AP1	cyclin-dependent kinase 2 associated protein 1	-1.52	0.0051	0.436
NAT10	N-acetyltransferase 10 (GCN5-related)	-1.53	0.0100	0.420
POLD1	polymerase (DNA directed), delta 1, catalytic subunit	-1.53	0.0366	0.465
LOC100626307	DNA primase large subunit-like	-1.53	0.0347	0.465
EIF4E	eukaryotic translation initiation factor 4E	-1.53	0.0080	0.419
MRTO4	MRT4 homolog, ribosome maturation factor	-1.53	0.0202	0.431
NPM3	nucleophosmin/nucleoplasmin 3	-1.53	0.0060	0.426
SLC25A11	solute carrier family 25 (mitochondrial carrier; oxoglutarate carrier), member 11	-1.54	0.0099	0.423
NIP7	NIP7, nucleolar pre-rRNA processing protein	-1.54	0.0051	0.439
OLR1	oxidized low density lipoprotein (lectin-like) receptor 1	-1.54	0.0046	0.433
IMPDH2	IMP (inosine 5-monophosphate) dehydrogenase 2	-1.54	0.0043	0.444
NACC1	nucleus accumbens associated 1, BEN and BTB (POZ) domain containing	-1.55	0.0168	0.435
PUS7	pseudouridylate synthase 7 (putative)	-1.55	0.0171	0.431
LOC100516390	ankyrin repeat domain-containing protein 26	-1.55	0.0150	0.429
HN1L	hematological and neurological expressed 1-like	-1.55	0.0216	0.436
LOC100739488	exportin-1-like	-1.55	0.0186	0.430
LOC100514269	phosphatidylinositol 4-phosphate 5-kinase type-1 alpha-like	-1.56	0.0284	0.452
PPP1R1A	protein phosphatase 1, regulatory (inhibitor) subunit 1A	-1.56	0.0328	0.459
SLC27A6	solute carrier family 27 (fatty acid transporter), member 6	-1.56	0.0184	0.429
FKBP4	FK506 binding protein 4, 59kDa	-1.56	0.0050	0.440
CTNNAL1	catenin (cadherin-associated protein), alpha-like 1	-1.56	0.0205	0.433
LOC100523881	focadhesin-like	-1.56	0.0347	0.464
SLC35B4	solute carrier family 35 (UDP-xylose/UDP-N-acetylglucosamine transporter), member B4	-1.56	0.0031	0.431
PSRC1	proline/serine-rich coiled-coil 1	-1.57	0.0159	0.434
PREP	prolyl endopeptidase	-1.57	0.0016	0.463
TEX12	testis expressed 12	-1.57	0.0367	0.465
LOC100523980	zinc finger protein 827	-1.57	0.0051	0.438
SNRPG	small nuclear ribonucleoprotein polypeptide G	-1.57	0.0137	0.436
TCP1	t-complex 1	-1.57	0.0030	0.432
LOC100626654	dipeptidyl peptidase 3	-1.58	0.0172	0.429
ARPC5L	actin related protein 2/3 complex, subunit 5-like	-1.58	0.0137	0.434
APOO	apolipoprotein O	-1.58	0.0116	0.426
TNFRSF11B	tumor necrosis factor receptor superfamily, member 11b	-1.58	0.0090	0.425
JUN	jun proto-oncogene	-1.58	0.0248	0.441
FANCM	Fanconi anemia, complementation group M	-1.59	0.0111	0.429
HAUS1	HAUS augmin-like complex, subunit 1	-1.59	0.0457	0.479
HINT3	histidine triad nucleotide binding protein 3	-1.59	0.0025	0.437
TUBA4A	tubulin, alpha 4a	-1.59	0.0009	0.564
MRPS28	mitochondrial ribosomal protein S28	-1.59	0.0109	0.431

XRCC2	X-ray repair complementing defective repair in Chinese hamster cells 2	-1.59	0.0443	0.478
LOC102161664	uncharacterized LOC102161664	-1.60	0.0038	0.439
DPH3	diphthamide biosynthesis 3	-1.60	0.0056	0.424
LOC100517579	olfactory receptor 4C46-like	-1.60	0.0111	0.429
CTPS1	CTP synthase 1	-1.60	0.0086	0.421
NAA40	N(alpha)-acetyltransferase 40, NatD catalytic subunit	-1.61	0.0080	0.420
GAR1	GAR1 homolog, ribonucleoprotein	-1.61	0.0155	0.430
PTPMT1	protein tyrosine phosphatase, mitochondrial 1	-1.61	0.0444	0.478
LOC106508523	breast cancer type 2 susceptibility protein-like	-1.62	0.0301	0.455
RFK	riboflavin kinase	-1.62	0.0012	0.471
LOC100738016	MKI67 FHA domain-interacting nucleolar phosphoprotein-like	-1.62	0.0068	0.422
CENPK	centromere protein K	-1.63	0.0441	0.478
RTN4IP1	reticulon 4 interacting protein 1	-1.63	0.0004	0.600
TOMM20	translocase of outer mitochondrial membrane 20 homolog (yeast)	-1.63	0.0065	0.435
PSMC3IP	PSMC3 interacting protein	-1.64	0.0174	0.429
ABCE1	ATP-binding cassette, sub-family E (OABP), member 1	-1.64	0.0120	0.430
NOP14	NOP14 nucleolar protein	-1.65	0.0045	0.436
NGFRAP1	nerve growth factor receptor (TNFRSF16) associated protein 1	-1.65	0.0450	0.478
DSCC1	DNA replication and sister chromatid cohesion 1	-1.67	0.0295	0.455
TUBA1B	tubulin, alpha 1b	-1.68	0.0228	0.435
SNRPA1	small nuclear ribonucleoprotein polypeptide A	-1.68	0.0011	0.497
SNRPA	small nuclear ribonucleoprotein polypeptide A	-1.68	0.0086	0.421
UBE2N	ubiquitin-conjugating enzyme E2N	-1.68	0.0070	0.421
TUBB	tubulin, beta class I	-1.69	0.0167	0.437
KRT18	keratin 18, type I	-1.69	0.0195	0.429
GFM2	G elongation factor, mitochondrial 2	-1.70	0.0014	0.447
HELLS	helicase, lymphoid-specific	-1.72	0.0170	0.432
HSPH1	heat shock 105kDa/110kDa protein 1	-1.72	0.0449	0.478
RAB34	RAB34, member RAS oncogene family	-1.72	0.0125	0.429
LOC100622080	decaprenyl-diphosphate synthase subunit 1-like	-1.73	0.0050	0.440
CCNE1	cyclin E1	-1.73	0.0267	0.448
AMD1	adenosylmethionine decarboxylase 1	-1.74	0.0164	0.435
C14H1orf131	chromosome 14 open reading frame, human C1orf131	-1.75	0.0037	0.439
RFC4	replication factor C (activator 1) 4, 37kDa	-1.75	0.0222	0.436
HSPA4L	heat shock 70kDa protein 4-like	-1.76	0.0234	0.438
LOC100526069	testis-expressed sequence 30 protein	-1.76	0.0052	0.427
RAD51	RAD51 recombinase	-1.77	0.0412	0.473
TOMM40	translocase of outer mitochondrial membrane 40 homolog (yeast)	-1.77	0.0025	0.441
CDO1	cysteine dioxygenase type 1	-1.77	0.0441	0.478
SNRPF	small nuclear ribonucleoprotein polypeptide F	-1.79	0.0130	0.432
MCM4	minichromosome maintenance complex component 4	-1.79	0.0412	0.472
FAM169A	family with sequence similarity 169, member A	-1.80	0.0086	0.422
LOC100624445	gamma-glutamylcyclotransferase-like	-1.80	0.0222	0.437
LOC100737936	dynamin-1-like protein	-1.80	0.0017	0.459
LRRC59	leucine rich repeat containing 59	-1.81	0.0461	0.479
CHAF1B	chromatin assembly factor 1, subunit B (p60)	-1.81	0.0226	0.437
DDIAS	DNA damage-induced apoptosis suppressor	-1.81	0.0298	0.456
TIPIN	TIMELESS interacting protein	-1.82	0.0155	0.430
SNRPD1	small nuclear ribonucleoprotein D1 polypeptide 16kDa	-1.82	0.0320	0.459
LOC100621671	ectonucleotide pyrophosphatase/phosphodiesterase family member 3	-1.85	0.0180	0.429
LOC100153906	DNA replication ATP-dependent helicase/nuclease DNA2	-1.86	0.0312	0.457
LOC100738210	bis(5-adenosyl)-triphosphatase ENPP4	-1.86	0.0140	0.432
CCL2	chemokine (C-C motif) ligand 2	-1.91	0.0277	0.448
SLC16A6	solute carrier family 16, member 6	-1.91	0.0006	0.630
CYCS	cytochrome c, somatic	-1.92	0.0005	0.577

LOC100154105	kelch repeat and BTB domain-containing protein 6	-1.94	0.0103	0.423
BFSP1	beaded filament structural protein 1, filensin	-1.95	0.0467	0.479
LYAR	Ly1 antibody reactive	-1.95	0.0245	0.441
LOC102163415	T-complex protein 1 subunit zeta	-1.96	0.0082	0.418
SLC51A	solute carrier family 51, alpha subunit	-1.96	0.0476	0.480
RANBP1	RAN binding protein 1	-1.97	0.0054	0.431
ATP6V0D2	ATPase, H+ transporting, lysosomal 38kDa, V0 subunit d2	-2.00	0.0198	0.429
NDUFAF4	NADH dehydrogenase (ubiquinone) complex I, assembly factor 4	-2.00	0.0026	0.429
SKA1	spindle and kinetochore associated complex subunit 1	-2.00	0.0331	0.459
MUC13	mucin 13, cell surface associated	-2.02	0.0180	0.429
DNAJC9	DnaJ (Hsp40) homolog, subfamily C, member 9	-2.06	0.0200	0.429
TUBA1C	tubulin, alpha 1c	-2.06	0.0065	0.432
ENPP4	ectonucleotide pyrophosphatase/phosphodiesterase 4 (putative)	-2.08	0.0061	0.424
OSGIN2	oxidative stress induced growth inhibitor family member 2	-2.09	0.0171	0.431
ARL2BP	ADP-ribosylation factor-like 2 binding protein	-2.14	0.0010	0.555
STEAP2	STEAP family member 2, metalloreductase	-2.16	0.0461	0.479
LOC100623233	PCNA-associated factor	-2.24	0.0447	0.478
SLC51B	solute carrier family 51, beta subunit	-2.28	0.0123	0.430
GINS1	GINS complex subunit 1 (Psf1 homolog)	-2.38	0.0260	0.447
WBP5	WW domain binding protein 5	-2.44	0.0376	0.466
UQCR10	ubiquinol-cytochrome c reductase complex 7.2 kDa protein	-3.02	0.0216	0.436
LOC100511841	UDP-glucuronosyltransferase 1-10	-3.38	0.0093	0.424

*Filter criteria: FC > 1.3 and < -1.3 and $P < 0.05$ between SINS high vs. SINS low. FCs were calculated from the signal log ratios, which were calculated from n = 6 microarrays per group.

Table S3 Plasma concentrations of amino acids in suckling piglets of the SINS high group and the SINS low group

	SINS low	SINS high
	µmol/L	
Alanine	466.2 ± 157.3	494.7 ± 108.5
Arginine	152.7 ± 22.84	144.1 ± 53.56
Asparagine	44.1 ± 30.2	58.4 ± 24.0
Aspartate	13.0 ± 3.59	11.6 ± 1.42
Citrulline	59.5 ± 12.3	69.8 ± 28.8
Glutamine	520.8 ± 139.7	428.8 ± 76.27
Glutamate	106.8 ± 27.57	86.0 ± 43.2
Glycine	875.5 ± 268.6	1037.3 ± 233.5
Histidine	98.2 ± 19.6	106.1 ± 40.74
Isoleucine	130.3 ± 18.16	120.9 ± 48.97
Leucine	233.5 ± 90.52	202.2 ± 61.42
Lysine	166.0 ± 30.96	154.7 ± 33.72
Methionine	37.2 ± 5.62	39.3 ± 17.4
Ornithine	63.1 ± 16.0	78.6 ± 11.7[#]
Phenylalanine	81.4 ± 23.4	100.4 ± 33.2
Proline	461.5 ± 141.9	490.7 ± 130.2
Serine	160.8 ± 68.6	230.1 ± 118.5
Threonine	168.5 ± 49.0	165.8 ± 66.8
Tryptophan	66.5 ± 13.9	48.6 ± 20.6
Tyrosine	109.1 ± 53.4	140.5 ± 78.6
Valine	356.2 ± 103.1	324.3 ± 74.9

Data are means ± SD for n = 6 piglets/group. *$P < 0.05$; $^{\#}P < 0.1$

Table S4 Plasma concentrations of biogenic amines and other amino acid metabolites in suckling piglets of the SINS high group and the SINS low group

	SINS low	SINS high
	µmol/L	
Asymmetric dimethylarginine	1.07 ± 0.33	1.12 ± 0.21
Alpha-aminoadipic acid	12.8 ± 6.81	11.9 ± 9.78
Carnosine	12.7 ± 3.11	16.7 ± 3.96[#]
Creatinine	114.1 ± 46.5	93.6 ± 59.3
Histamine	0.34 ± 0.30	0.50 ± 0.46
Kynurenine	1.28 ± 1.56	1.21 ± 1.25
Methionine-sulfoxide	2.49 ± 0.66	3.60 ± 0.96*
Putrescine	0.46 ± 0.17	1.01 ± 0.81
Sarcosine	2.16 ± 0.53	3.04 ± 1.40
Symmetric dimethylarginine	0.50 ± 0.28	0.96 ± 0.62
Serotonin	0.10 ± 0.05	0.14 ± 0.10
Spermidine	0.21 ± 0.01	0.24 ± 0.05
Spermine	0.16 ± 0.01	0.17 ± 0.03
trans-4-hydroxyproline	39.4 ± 16.5	63.8 ± 32.7
Taurine	62.4 ± 18.2	91.8 ± 31.4[#]

Data are means ± SD for n = 6 piglets/group. *$P < 0.05$; [#]$P < 0.1$

Table S5 Plasma concentrations of carnitine and acylcarnitines in suckling piglets of the SINS high group and the SINS low group

	SINS low	SINS high
	µmol/L	
Carnitine (free), C0	17.12 ± 3.55	15.40 ± 5.35
Acetylcarnitine, C1	1.79 ± 0.79	2.77 ± 2.13
Propionylcarnitine, C3	0.21 ± 0.15	0.18 ± 0.05
Hydroxybutyrylcarnitine, C4-OH	0.10 ± 0.02	0.09 ± 0.01
Butyrylcarnitine, C4	0.091 ± 0.057	0.093 ± 0.046
Tetradecenoylcarnitine, 14:1	0.034 ± 0.018	0.050 ± 0.034

Data are means ± SD for n = 6 piglets/group.

Table S6 Plasma concentrations of lysophosphatidylcholine (LPC) species in suckling piglets of the SINS high group and the SINS low group

	SINS low	SINS high
	µmol/L	
lysoPC a C16:0	33.93 ± 11.09	29.88 ± 6.54
lysoPC a C16:1	1.41 ± 0.25	1.87 ± 0.67
lysoPC a C17:0	1.11 ± 0.60	0.91 ± 0.45
lysoPC a C18:0	22.33 ± 8.22	20.25 ± 3.70
lysoPC a C18:1	17.27 ± 3.65	21.57 ± 9.29
lysoPC a C18:2	18.42 ± 4.85	19.42 ± 9.53
lysoPC a C20:3	1.33 ± 0.39	1.63 ± 0.53
lysoPC a C20:4	4.61 ± 1.05	5.59 ± 1.59
lysoPC a C26:0	0.082 ± 0.028	0.081 ± 0.031
lysoPC a C26:1	0.058 ± 0.027	0.061 ± 0.017
lysoPC a C28:1	0.149 ± 0.057	0.145 ± 0.057

Data are means ± SD for n = 6 piglets/group.

Table S7 Plasma concentrations of sphingomyelin (SM) and hydroxy sphingomyelin (SM (OH)) species in suckling piglets of the SINS high group and the SINS low group

	SINS low	SINS high
	µmol/L	
SM C16:0	39.95 ± 6.17	40.73 ± 1.25
SM C16:1	7.03 ± 2.24	6.64 ± 0.54
SM C18:0	13.08 ± 3.04	14.20 ± 1.93
SM C18:1	3.79 ± 0.96	4.16 ± 0.83
SM C20:2	0.52 ± 0.11	0.65 ± 0.19
SM C22:3	2.95 ± 0.83	3.85 ± 0.91
SM C24:0	11.04 ± 3.79	9.38 ± 1.26
SM C24:1	12.29 ± 3.70	10.96 ± 1.70
SM C26:0	0.12 ± 0.05	0.10 ± 0.03
SM C26:1	0.34 ±0.15	0.29 ± 0.03
SM (OH) C14:1	2.86 ± 0.52	3.63 ± 1.20
SM (OH) C16:1	3.02 ± 0.96	3.19 ± 0.67
SM (OH) C22:1	4.53 ± 2.08	2.92 ± 1.49
SM (OH) C22:2	2.11 ± 0.65	1.89 ± 0.43
SM (OH) C24:1	0.79 ± 0.21	0.87 ± 0.15

Data are means ± SD for n = 6 piglets/group. *P < 0.05; $^{\#}P$ < 0.1

Table S8 Plasma concentrations of diacyl (aa) and acyl-alkyl (ae) phosphatidylcholine (PC) species in suckling piglets of the SINS high group and the SINS low group

	SINS low	SINS high
	µmol/L	
PC aa C28:1	0.87 ± 0.16	1.14 ± 0.38
PC aa C30:0	2.24 ± 0.50	3.06 ± 1.40
PC aa C30:2	0.44 ± 0.18	0.42 ± 0.09
PC aa C32:0	14.12 ± 11.01	27.46 ± 18.66
PC aa C32:1	7.01 ± 4.25	13.12 ± 7.57
PC aa C32:2	1.36 ± 0.33	2.09 ± 0.88
PC aa C32:3	0.25 ± 0.04	0.26 ± 0.06
PC aa C34:1	64.37 ± 10.08	80.98 ± 18.50
PC aa C34:2	71.90 ± 12.15	79.85 ± 13.43
PC aa C34:3	11.10 ± 2.92	13.94 ± 4.02
PC aa C34:4	0.63 ± 0.19	1.00 ± 0.39
PC aa C36:0	7.17 ± 2.92	11.09 ± 5.46
PC aa C36:1	46.53 ± 7.13	57.15 ± 12.90
PC aa C36:2	69.32 ± 12.33	89.10 ± 28.01
PC aa C36:3	38.57 ± 8.50	51.23 ± 15.46
PC aa C36:4	43.5 ± 7.01	50.9 ± 6.48[#]
PC aa C36:5	16.81 ± 4.38	19.68 ± 4.44
PC aa C36:6	0.66 ± 0.19	1.05 ± 0.31*
PC aa C38:0	0.89 ± 0.32	1.38 ± 0.66
PC aa C38:1	1.28 ± 0.50	1.29 ± 0.19
PC aa C38:3	29.33 ± 7.87	32.85 ± 2.89
PC aa C38:4	51.45 ± 8.15	55.93 ± 3.00
PC aa C38:5	40.58 ± 7.75	43.90 ± 2.30
PC aa C38:6	29.3 ± 4.49	36.3 ± 6.57[#]
PC aa C40:2	0.30 ± 0.11	0.37 ± 0.07
PC aa C40:3	0.37 ± 0.10	0.66 ± 0.24*
PC aa C40:4	7.36 ± 3.55	7.08 ± 3.78
PC aa C40:5	29.77 ± 11.80	26.08 ± 9.81
PC aa C40:6	31.82 ± 7.07	33.67 ± 2.91

PC aa C42:0	0.12 ± 0.04	0.14 ± 0.03
PC aa C42:1	0.14 ± 0.04	0.17 ± 0.05
PC aa C42:4	0.23 ± 0.09	0.30 ± 0.13
PC aa C42:5	0.42 ± 0.32	0.74 ± 0.50
PC aa C42:6	0.91 ± 0.22	1.26 ± 0.65
PC ae C30:0	0.32 ± 0.15	0.62 ± 0.37$^{\#}$
PC ae C30:1	0.23 ± 0.07	0.36 ± 0.15$^{\#}$
PC ae C32:1	1.71 ± 0.57	2.90 ± 1.46$^{\#}$
PC ae C32:2	0.61 ± 0.42	1.06 ± 0.56
PC ae C34:0	1.28 ± 0.61	2.48 ± 1.63
PC ae C34:1	8.20 ± 2.27	13.21 ±4.91*
PC ae C34:2	7.26 ± 1.54	8.38 ± 1.35
PC ae C34:3	3.28 ± 0.69	3.19 ± 0.54
PC ae C36:0	0.52 ± 0.13	0.87 ± 0.36$^{\#}$
PC ae C36:1	8.66 ± 3.15	11.24 ± 3.26
PC ae C36:2	10.71 ± 3.25	13.92 ± 4.06
PC ae C36:3	4.17 ± 0.84	5.80 ± 1.67$^{\#}$
PC ae C36:4	3.95 ± 0.59	5.20 ±1.42$^{\#}$
PC ae C36:5	2.87 ± 1.25	3.71 ± 1.67
PC ae C38:0	1.48 ± 0.38	2.23 ± 0.67*
PC ae C38:1	0.96 ± 0.41	1.23 ± 0.29
PC ae C38:2	2.08 ± 0.82	2.59 ± 0.79
PC ae C38:3	2.99 ± 1.21	3.80 ± 1.06
PC ae C38:4	6.78 ± 2.24	8.12 ± 2.54
PC ae C38:5	5.23 ± 1.09	6.94 ± 1.62$^{\#}$
PC ae C38:6	2.14 ± 1.39	3.28 ± 1.97
PC ae C40:1	0.87 ± 0.31	1.53 ± 0.78$^{\#}$
PC ae C40:2	0.77 ± 0.31	0.75 ± 0.14
PC ae C40:3	1.14 ± 0.74	1.11 ± 0.62
PC ae C40:4	1.81 ± 0.92	1.89 ± 0.85
PC ae C40:5	3.70 ± 1.63	3.61 ± 1.35
PC ae C40:6	3.03 ± 0.77	3.66 ± 0.69
PC ae C42:1	0.50 ± 0.15	0.62 ± 0.15
PC ae C42:2	0.49 ± 0.21	0.63 ± 0.11

PC ae C42:3	0.37 ± 0.10	0.66 ± 0.30*
PC ae C44:3	0.12 ± 0.04	0.13 ± 0.03
PC ae C44:5	0.25 ± 0.03	0.30 ± 0.08
PC ae C44:6	0.17 ± 0.04	0.15 ± 0.03
Sum PC aa	621.1 ± 98.6	745.7 ± 123.9#
Sum PC ae	88.6 ± 20.2	116.1 ± 26.3#
Sum PC aa+ae	709.7 ± 118.7	861.8 ± 147.0#

Data are means ± SD for n = 6 piglets/group. *$P < 0.05$; #$P < 0.1$
